# Supplementary material for: Decoupling excitons from high-frequency vibrations in organic molecules
Source: Nature. 2024 May 8;629(8011):355–62. doi: 10.1038/s41586-024-07246-x (PMC11078737; doi:10.1038/s41586-024-07246-x)
Supplement: Supplementary file 1 — The file contains synthesis and characterization, Supplementary experimental data, quantum chemical calculations, and an in-depth discussion regarding the theory of non-radiative loss and vibrational coherence. [file 41586_2024_7246_MOESM1_ESM.pdf]

nature portfolio

https://doi.org/10.1038/s41586-024-07246-x

Supplementary information

Decoupling excitons from high-frequency vibrations in organic molecules

In the format provided by the authors and unedited

Supplementary Information for

Decoupling excitons from high-frequency vibrations in organic molecules

Pratyush Ghosh[1], Antonios M. Alvertis[2,3], Rituparno Chowdhury[1], Petri Murto[4], Alexander J. Gillett[1], Shengzhi Dong[5], Alexander J. Sneyd[1], Hwan-Hee Cho[1], Emrys W Evans[1,6], Bartomeu Monserrat[1,7], Feng Li[5], Christoph Schnedermann[1], Hugo Bronstein[1,4], Richard H. Friend[1], Akshay Rao[1*]

Affiliation:

[1]Cavendish Laboratory, University of Cambridge, Cambridge, UK

[2]KBR, Inc, NASA Ames Research Center, Moffett Field, California 94035, United States

[3]Materials Sciences Division, Lawrence Berkeley National Laboratory, Berkeley, California, USA

[4]Yusuf Hamied Department of Chemistry, University of Cambridge, Cambridge, UK

[5]State Key Laboratory of Supramolecular Structure and Materials, College of Chemistry, Jilin University, Changchun, China

[6]Department of Chemistry, Swansea University, Singleton Park, Swansea, UK

[7]Department of Materials Science and Metallurgy, University of Cambridge, Cambridge, UK

[*]Author to whom correspondence should be addressed; E-mail: ar525@cam.ac.uk

Table of Contents

Section 1: Materials synthesis and characterization…………………………………………….…4

Section 2: Off-resonant steady state Raman spectroscopy ………………………………......7

Section 3: Temporal characterization and correction of optical pulses

3.1: Effective Time Resolution……………………………………………………………...8

3.2: Time Resolution correction……………………………………………………………9

3.3: Effective Time Resolution for the band selective resolution………………….......9

Section 4: Coherent oscillation maps of representative radical, TADF and NFA………...10

Section 5: Probe wavelength-resolved analysis of the resonant IVS

5.1: APDC-DTPA…………………………………………………….……………10

5.2: TTM-3PCz…………………………………………………………………………..12

Section 6: Assignment of the excited-state vibrational coherence based on phase analysis of the coherent oscillations……………………………………………………………13

Section 7: Excited-state impulsive vibrational spectroscopy of 4CzIPN in 3-pulse geometry……………………………………………………………………………………..14

Section 8: Ultrafast dynamics of TTM-TPA (Band selective excitation) ……….……...16

Section 9: Analysis of the low frequency vibrational modes of TTM-3PCz.………………17

Section 10: Charge-transfer character……………………………………………………..17

Section 11: Electronic structure Calculation of TTM-TPA…………………………………..18

Section 12: Calculation of exciton-vibration interactions in studied molecules…………..18

Section 13: Excited state energies along a pair of high-frequency normal modes……….20

Section 14: Molecular orbital (MO)-vibrational coupling

14.1: Electronic localisation of the HOMO of non-planar donor moieties …………..22

14.2: Electronic localisation of the HOMO of APDC-DTPA and TTM-TPA ……….26

14.3: Electronic localisation of the SOMO orbital of TTM-Donor radicals ……….. 26

14.4: Electronic localisation and vibrational decoupling ……………………………..28

Section 15: Zero-point renormalisation of excited state energies using Monte Carlo sampling ………………………………………………………………………………….…31

Section 16: Summary of the efficient red LEDs ……………………………………………..33

Section 17: Synthesis of M2TTM-3PCz and M2TTM-2PCz ……………………...……..33

Section 18: mode-resolved Non-radiative loss probed by vibrational coherence ...……..42

Section 19: Impulsive vibration spectroscopy of TTM-TPA in variable solvent polarity....47

Section 20: Extended discussion on dependence of exciton-vibration coupling on degree of charge transfer character...50

Section 21: Extended discussion on vibrational coupling and non-radiative decay in non-fullerene acceptors...51

References………………………………………………………………………………………..54

Section 1: **Materials synthesis and characterizations**

General information: All initial materials and solvents used were purchased from commercial supplier and used without further purification, unless otherwise stated. Column chromatography was performed using silica gel (200-300 mesh). The concentration of sample solutions used for the optical measurements is around 10^{-5} mol/L.

The ^{1}H-NMR spectrum was measured with a Bruker AVANCZIII 500 spectrometer at 500 MHz with tetramethylsilane (TMS) as the internal standard, using deuterated acetone (*d*-acetone) as solvent at 298 K. GC-MS mass spectra of all compounds were recorded on a Thermo Fisher ITQ1100 mass spectrometer. IR spectra of radical was recorded with Brucker VERTEX 80V. EPR spectrum of the radical was recorded on a Bruker ELEXSYSII E500 CW-EPR spectrometer.

Synthesis: The HTTM was prepared as reported[1,2] (Scheme 1) (**GC-MS (m/z):** HTTM calculated for $C_{19}H_7Cl_9$, 554.32; found, 553.65) and TPA pinacole borane was purchased from commercially available source. The stepwise synthetic method for the TTM-TPA radical is outlined in Scheme 2.

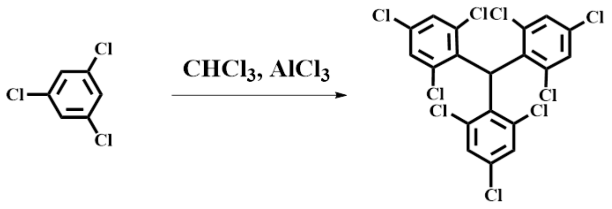

Scheme 1. Synthesis of the HTTM

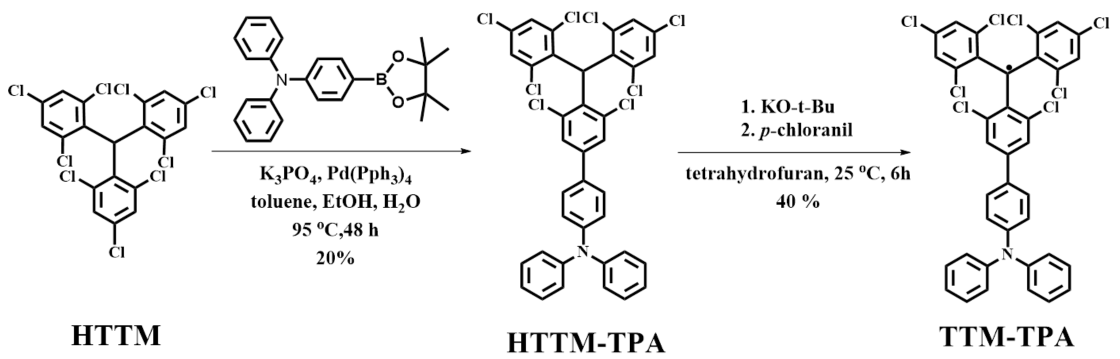

Scheme 2. Synthesis of the TTM-TPA radical

(1) Synthesis of HTTM-TPA

HTTM (1.0 g, 1.80 mmol) and the TPA pinacole borane (0.67 g, 1.80 mmol) was dissolved in a mixed solvent of toluene (12 ml), K_2PO_3 aqueous solution (8 ml, 2 mol / L) and ethanol (4 ml), and catalyst $Pd(Pph_3)_4$ (0.10 g, 0.09 mmol) was added under argon atmosphere. The mixture was stirred at 95℃ for 48 h under argon atmosphere and in the dark. After the reaction mixture cooling to room temperature, the solution was extracted with dichloromethane, organic layer was collected and dried. The solvent was removed under vacuum and the crude product was purified by silica gel column chromatography (using petroleum ether: dichloromethane = 10:1 v/v). HTTM-TPA was obtained as a white solid. **GC-MS (m/z):** calculated for $C_{37}H_{21}Cl_8N$, 763.18; found, 762.59; **^{1}H NMR** (500 MHz, *d*-acetone) δ 7.80 (d, *J* = 1.9 Hz, 1H), 7.71 (d, *J* = 8.7 Hz, 2H), 7.68-7.61 (m, 3H), 7.48 (t, *J* = 2.6 Hz, 2H), 7.41-7.31 (m, 4H), 7.22-7.06 (m, 8H), 6.84 (s, 1H).

(2) Synthesis of TTM-TPA

Under argon atmosphere and in the dark, the HTTM-TPA (1.00 equiv) was dissolved in dry THF (40 ml). Then KO^tBu (4.00 equiv) was added, the solution become claret-colored immediately. The solution was stirred for 2 h in the dark at room temperature, and then p-Chloranil (5 equiv) was added. The solution was stirred for further 3 h. After the reaction finished, the solvent was removed under vacuum and the crude product was purified by silica gel column chromatography (using petroleum ether: dichloromethane = 10:1v/v). The crude product was recrystallized twice from dichloromethane and methanol and a brown red solid was obtained. **GC-MS (m/z):** calculated for $C_{37}H_{20}Cl_8N$, 762.17; found, 761.83; **IR, EPR** and **TGA** spectra are shown below in Supplementary figure 1-2. Photoluminescence properties of the TTM-TPA solution (toluene) are shown in the Supplementary figure 3.

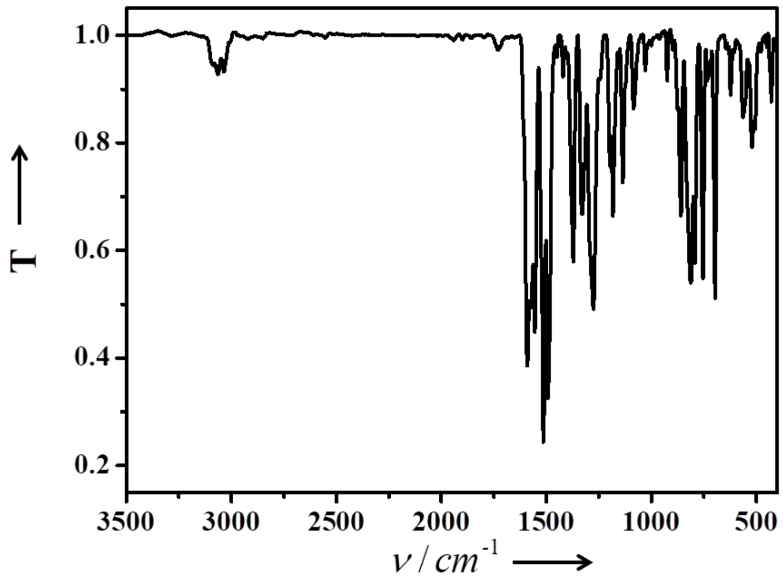

Supplementary Figure 1. IR spectrum of TTM-TPA

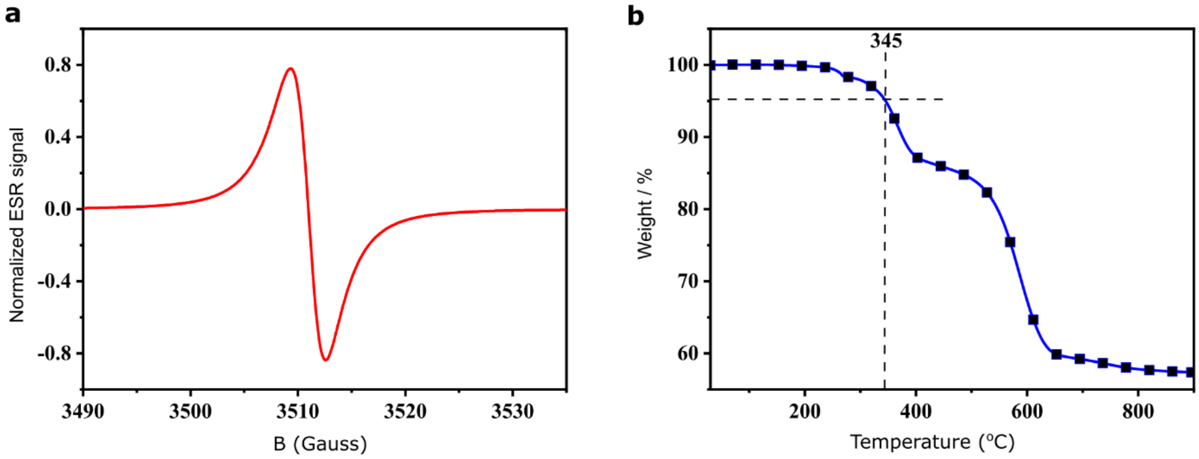

Supplementary Figure 2. Spin and thermal characterization of TTM-TPA a, EPR spectra for TTM-TPA (solid samples) at room temperature. ESR, electron spin resonance; *B*, magnetic field. **b,** Thermogravimetric analysis measurements show thermal decomposition temperatures of 345 °C (TTM-TPA)

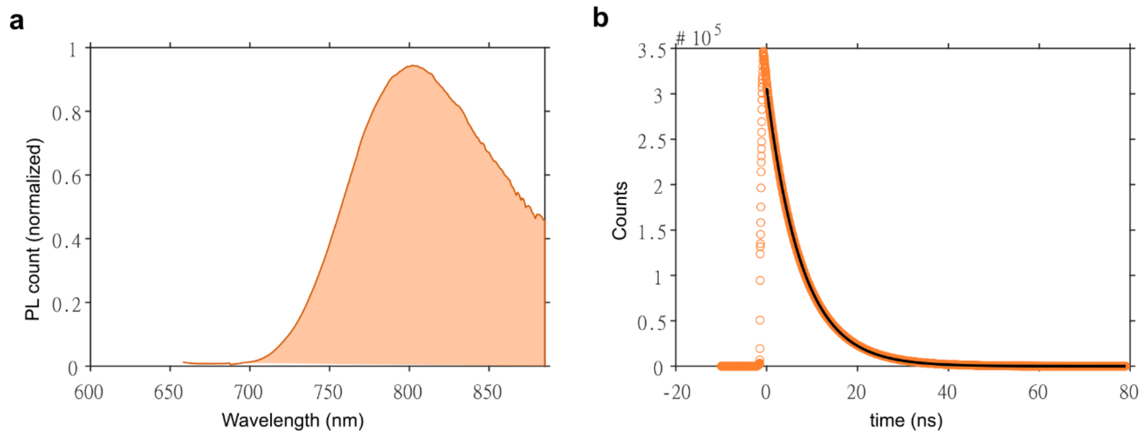

Supplementary Figure 3: Photoluminescence characterization: a, the steady-state PL spectra ($\lambda max = 800\ nm$) obtained for TTM-TPA (0.005mg/ml Toluene) while exciting at 350 nm. **b,** TCSPC decay kinetics showing the D_1 lifetime 8.4 ns

Section 2: **Off-resonant steady state Raman spectroscopy**

The off-resonant steady state Raman spectroscopy is performed on the investigated radical, TADF and representative NFA molecules. The photon energy of the incident laser beam is tuned below the optical absorption band of the studied systems. As can be visualised in the Supplementary Figure 4, the steady-state Raman spectra of TTM-3PCz has strong high-frequency modes at 1256, 1566, 1600, 1627 cm^{-1} along with the low-frequency mode at 225 cm^{-1} which is the most dominant mode in the experimental IVS spectra. Similarly, TADFs APDC-DTPA and 4CzIPN have a series of high-frequency Raman active modes at 1190, 1540, 1602 cm^{-1} and 1352, 1436, 1662 cm^{-1} respectively. The NFA, IO-4Cl has strong high-frequency modes at 1275, 1357, 1432, 1468, 1600 cm^{-1} in the steady-state Raman spectra which are also present with reasonable intensity in the IVS spectra having frequencies 1270, 1368, 1422, 1470 and 1596 cm^{-1} (see Extended Figure 2). This result suggests that the radical and TADFs studied feature Raman active high-frequency vibrational modes and the suppression of the intensity of these modes is a signature of the electronic transition which is involved in the resonant impulsive transition.

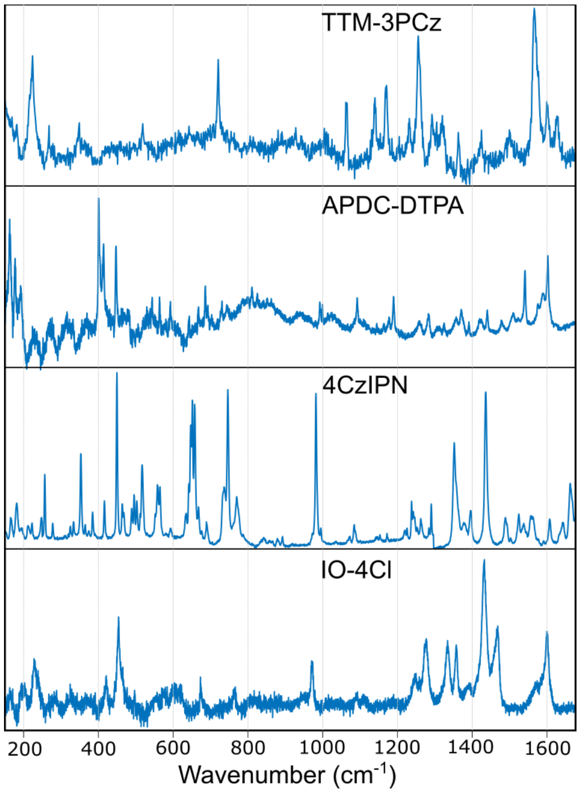

Supplementary Figure 4: Off-resonant steady state Raman spectra of representative radical (TTM-3PCz), TADF (APDC-DTPA, 4CzIPN) and NFA (IO-4Cl) molecules. Experient is performed on TTM-3PCz (powder), APDC-DTPA (powder), IO-4Cl (spin-coated neat film) with 785 nm laser and on 4CzIPN (evaporated neat film) with 633 nm laser.

Section 3: **Temporal characterization and correction of optical pulses**

3.1. Effective Time Resolution

As our detection scheme is spectrally-resolved, the time resolution (τ_{probe}) of the probe pulse is the transform limit which turns out to be ~4 fs for the 1030 nm seeded white light continuum[3]. The temporal profile retrieved from SHG-FROG shown in the Supplementary Figure 5, depicts the time resolution of the pump (τ_{pump}) to be 8.8 fs. Therefore, the time-resolution for the overall experiment in combination of pump and probe pulse is, $\tau_{res} = \sqrt{(\tau_{\text{probe}}^2 + \tau_{\text{pump}}^2)} = 9.67\ fs$.

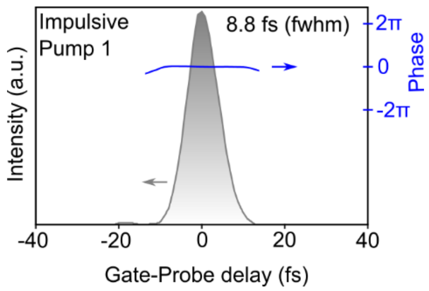

Supplementary Figure 5: Temporal profile of the impulsive pump 1 retrieved with SHG-FROG (pump pulse used for the all experiments reported in Fig. 2 in main text).

With the overall time resolution (τ_{res}) of 9.67 fs, we are theoretically able to impulsively excite and resolve vibrational modes up to ~3450 cm^{-1}.

3.2 Time Resolution Correction

The oscillatory signal recorded as a vibrational coherence in the time-domain is convolved with the intrinsic time resolution of the experiment. As a result, low frequency modes with longer oscillatory period are overemphasised in the frequency domain with respect to the high-frequency modes with shorter oscillatory period. To obtain the precise intensities we have employed a time-resolution correction method to account for the non-zero time-resolution (τ_{res}) of the experiment as previously reported[4].

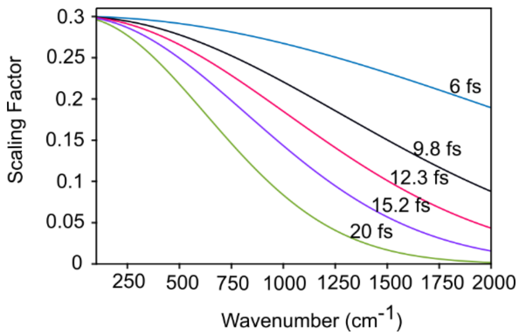

Supplementary Figure 6: **Scaling Factor computed against the vibrational mode frequencies for different time resolution:** 9.8 fs (black) corresponds to the resonant experiments reported in the Fig. 2 (main text); 12.3 fs (magenta), 15.2 fs (purple) corresponds to the P_1 and P_2 excitations in the experiment reported in the band-selective IVS experiment (Fig. 3, main text)

Briefly, to model the effect of the τ_{res} on an oscillation with frequency ω, we convolve a particular oscillation with a gaussian profile with full width half maxima of τ_{res}. Fast Fourier transformation (FFT) is performed on both the pristine and convolved oscillation and a scaling factor is obtained. The mode-resolved scaling factor for all frequencies of interest (100-2000 cm^{-1}) can be generated by simulating this method which is plotted in Supplementary Figure 6 including all the experiments performed in this study.

3.3 Effective Time Resolution for the band selective resolution

As visualised in retrieved FROG traces shown in Extended Data Fig. 6a, the time resolution of the pump (τ_{pump}) are 14.6 fs and 11.6 fs for the P_2 (D_2-rich excitation) and P_1 (D_1-excitation) respectively. Therefore, the overall time-resolution (τ_{res}) become 15.2 fs (P_2) and 12.3 fs (P_1) as the same white light continuum is used as probe pulse for both experiments (see SI section 3.1). The dominance of high-frequency oscillation on the raw data (see Extended Data Fig. 6b) for the D_2-rich data excited with a slower pulse with comparison to D_1 data which is excited with faster P_1 pulse, support the data (Fig. 3) and interpretation presented in the main text.

Section 4: **Coherent oscillation maps of representative radical, TADF and NFA**

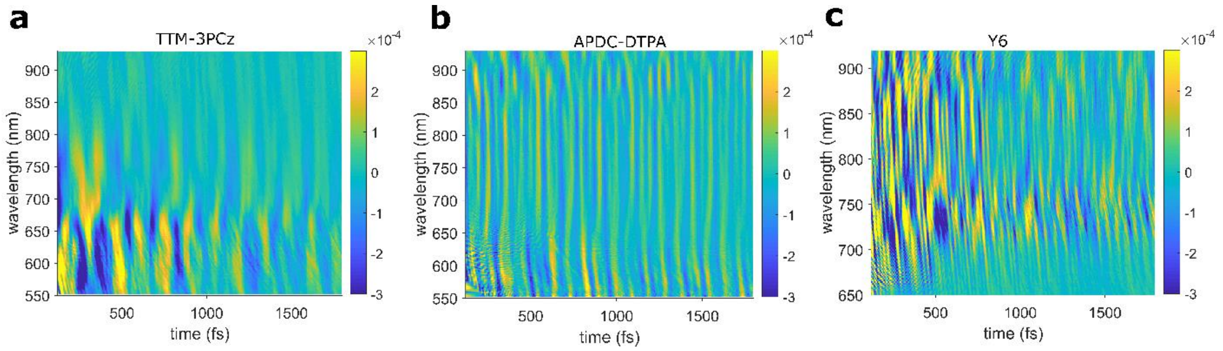

Supplementary Figure 7 Coherent oscillation maps of representative a, radical (TTM-3PCz), **b,** TADF (APDC-DTPA) and **c,** NFA (Y6). The maps after prepared by fitting and subsequently eliminating the electronic population dynamics from the pump-probe data at each probe wavelength.

Section 5: **Probe wavelength-resolved analysis of the resonant IVS**

5.1: APDC-DTPA

We remark that an alternative explanation for the absence of high-frequency modes in the excited state vibrational spectra of APDC-DTPA could be due to a lack of displacement between the charge-transfer S_1 state and the probed higher excited state(s), S_n (n≥2) which are involved in the PIA along the coordinates of the high-frequency modes.

The transient absorption spectra of APDC-DTPA in $CHCl_3$ (Supplementary Figure 8a) shows negative signal over the visible probe (530-940 nm) which can be attributed to the photo-induced absorption (PIA) of the initially excited CT S_1 state. As plotted in Supplementary Figure 8a, the photoluminescence spectra of APDC-DTPA spans from 650-800 nm which suggest the stimulated emission (SE) feature is convoluted within the TA spectrum (650-800 nm). Both the SE-rich PIA and pristine PIA share the same population kinetics (see Supplementary Figure 9) which indicates the origin of the SE and PIA from the same CT S_1 state. The early time (<2 ps) kinetics extracted from the SE+PIA (700-720 nm) and pristine PIA (880-900 nm) obtained from the resonant IVS are plotted in Supplementary Figure 8b, none of which shows fast oscillatory component (<30 fs) which corresponds to the carbon-carbon stretching modes. The Fourier transformed vibrational spectra are plotted in Supplementary Figure 8c and 8d, extracted from SE-rich PIA and pristine PIA respectively. Both spectra do not show strong coupling to the vibrational modes in the high-frequency regime.

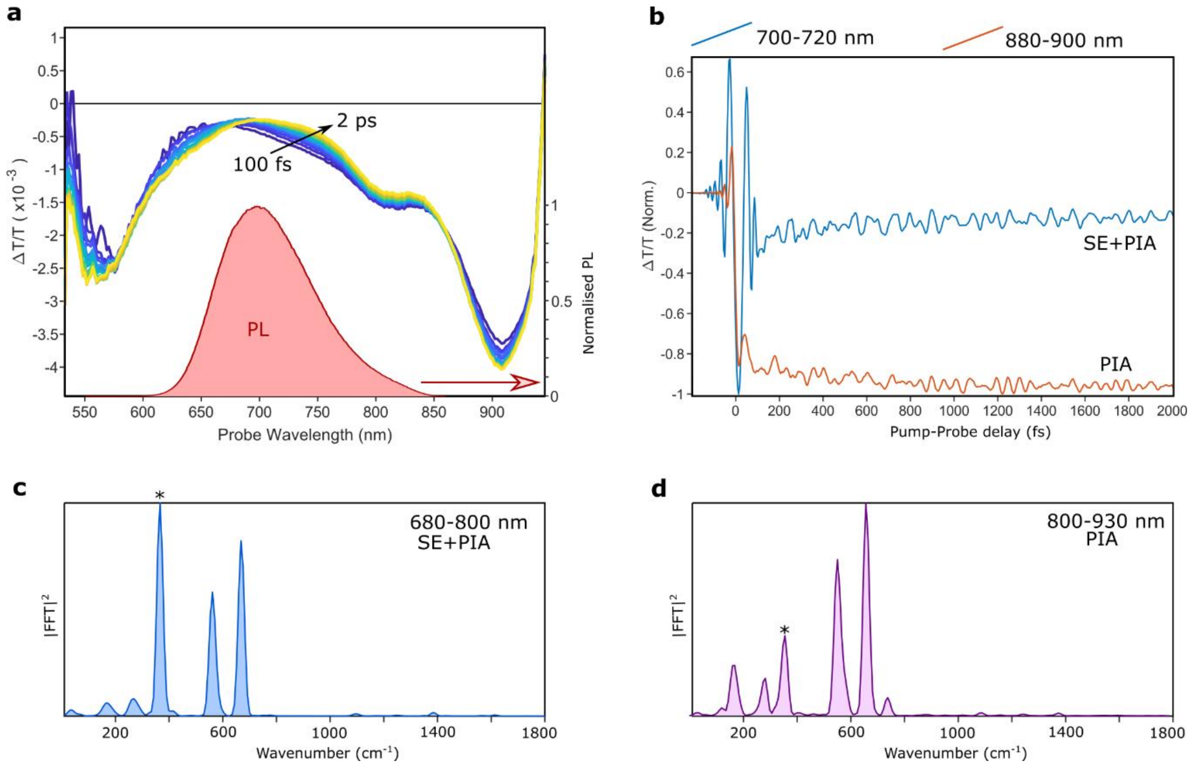

Supplementary Figure 8: **Probe wavelength-resolved analysis of the resonant IVS (pump-probe) of the APDC-DTPA: a,** Evolution of the transient absorption signal from 100fs to 2ps. The TA signals at each time steps are area normalized. The photoluminescence spectra of APDC-DTPA plotted in red area-plot. **b,** Temporal kinetic extracted from the 700-720 nm (SE-rich PIA) and 880-900 nm (pristine PIA). **c,** Fourier transformed vibrational spectra from the SE-rich (680-800 nm) transient absorption signal **d,** Fourier transformed vibrational spectra from the pristine PIA (800-930 nm) transient absorption signal. (Note: The Raman spectra presented on c-d panel are averaged over the probe wavelength in frequency domain after FFT to avoid artefacts that could arise in the time domain averaging due to phase-killing resulted from chirp artefact which could affect the intensity of high-frequency modes). The asterisk indicates $CHCl_3$ mode. SE: Stimulated Emission, PIA: Photo-induced emission.

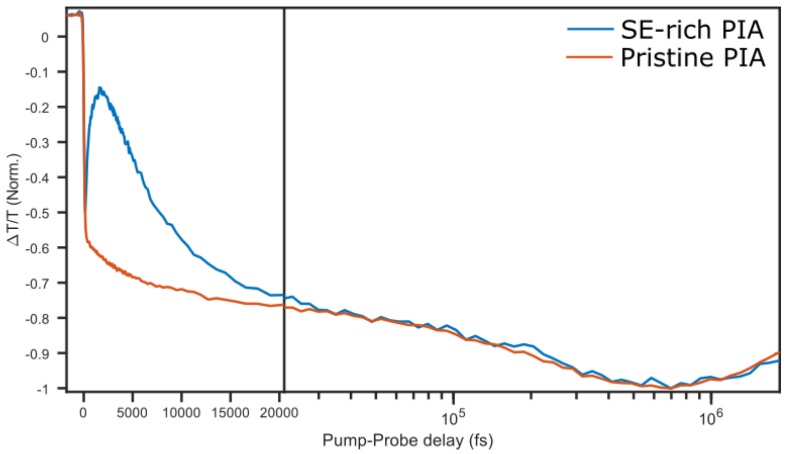

Supplementary Figure 9: **Long time (up to 2 ns) TA data of the APDC-DTPA ($CHCl_3$):** SE-rich kinetics obtained from the probe wavelength (670-690 nm) and the pristine PIA kinetics obtained for from the probe wavelength (880-900 nm). The mismatch in the ultrafast timescale (<20 ps) arises from the spectral shift happening in the SE-rich regime (see Supplementary Figure 5a). Both the traces share same kinetic (20 ps → 2 ns) which indicates both the features are arising from the same photoexcited species (S_1 CT)

5.2: TTM-3PCz

The absorption spectra of the charge-transfer band and the photoluminescence spectra of TTM-3PCz ($CHCl_3$) are plotted in Supplementary Figure 10a. The transient absorption spectra at different time delays (100 fs → 2 ps) are plotted in the Supplementary Figure 10b. From the absorption and photoluminescence spectra, it can be stated that the ground state bleach (GSB) and stimulated emission (SE) are convoluted with the TA spectrum for the wavelength span ~530-680 nm and 620-800 nm respectively. Supplementary Figure 10d-f visualises the Fourier transformed vibrational spectra extracted from the obtained vibrational coherence while probing PIA+GSB, PIA+SE and pristine PIA respectively. Similar like APDC-DTPA, no significant increase in the intensity of the Raman modes at high frequency regime moving from the pristine PIA to the SE-rich PIA. This confirms the nuclear wave packet motion generated on the D_1 potential energy surface after photoexcitation from D_0, is intrinsically decoupled from high-frequency modes and it is not arising from a non-displaced D_1 and D_n (n≥2) potential energy surface along the high frequency modes.

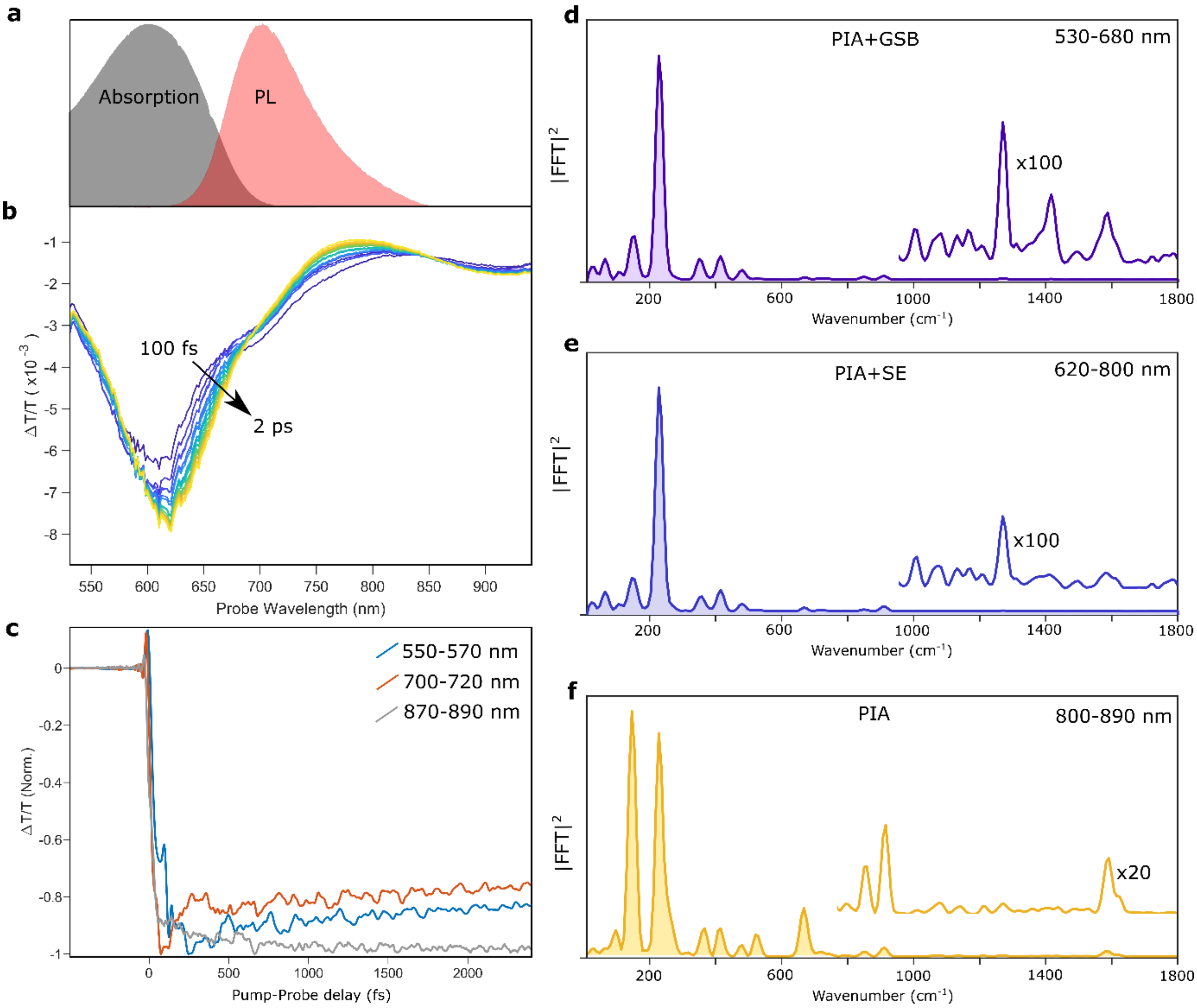

Supplementary Figure 10: **Probe wavelength-resolved analysis of the resonant IVS (pump-probe) of the TTM-3PCz: a,** the photoluminescence and absorption spectra of TTM-3PCz ($CHCl_3$) plotted in red and grey area-

plot respectively. **b,** Evolution of the transient absorption signal from 100 fs to 2 ps. The TA signals at each time steps are area normalized. **c,** Temporal kinetic extracted from the 700-720 nm (SE-rich PIA) and 880-900 nm (pristine PIA). **d,** Fourier transformed vibrational spectra extracted from the PIA+GSB (530-680 nm) convoluted transient absorption signal. **e,** Fourier transformed vibrational extracted spectra from the PIA+SE (620-800 nm) convoluted transient absorption signal. **f,** Fourier transformed vibrational extracted spectra from the pristine-PIA (800-890 nm) convoluted transient absorption signal. (Note: The Raman spectra presented on d-f panel are averaged over the probe wavelength in frequency domain after FFT to avoid artefacts that could arise in the time domain averaging due to phase-killing resulted from chirp artefact which could affect the intensity of high-frequency modes)

Section 6: **Assignment of the excited-state vibrational coherence based on phase analysis of the coherent oscillations**

In broadband resonant IVS, a nuclear wavepacket motion is generated after projecting the ground-state population into the excited-state. When the excited electronic state is displaced with respect to the ground electronic state along a normal mode coordinate, the wavepacket motion is launched at one side of excited potential energy surface and its motion to the other side of the potential energy surface along the normal mode coordinate is detected as vibrational coherence at each probe wavelength. The vibrational coherence generated on the displaced excited state surface shows a phase shift at a particular probe wavelength which corresponds to the minima of the potential energy surface[5]. Therefore, this phase shift of the excited state coherence which appears as a node in the coherent oscillation map corresponds closely to the photoluminescence maxima. As depicted in the Supplementary Figure 11a, the vibrational coherence is highly dominated by the low frequency mode with almost no contribution from fast high-frequency modes, has a node at 685 nm which nearly corresponds to the PL maxima (~700 nm). When an excited-state wavepacket is generated upon impulsive excitation, the effective energy-gap between ground and excited electronic states increases, which explains the slight blue shift of the node position with respect to the PL maxima[5]. Thus, this analysis additionally supports the high-frequency decoupled exciton-vibrational coupling for TTM-3PCz exciton. The schematic of this phenomenon is illustrated in Supplementary Figure 11b.

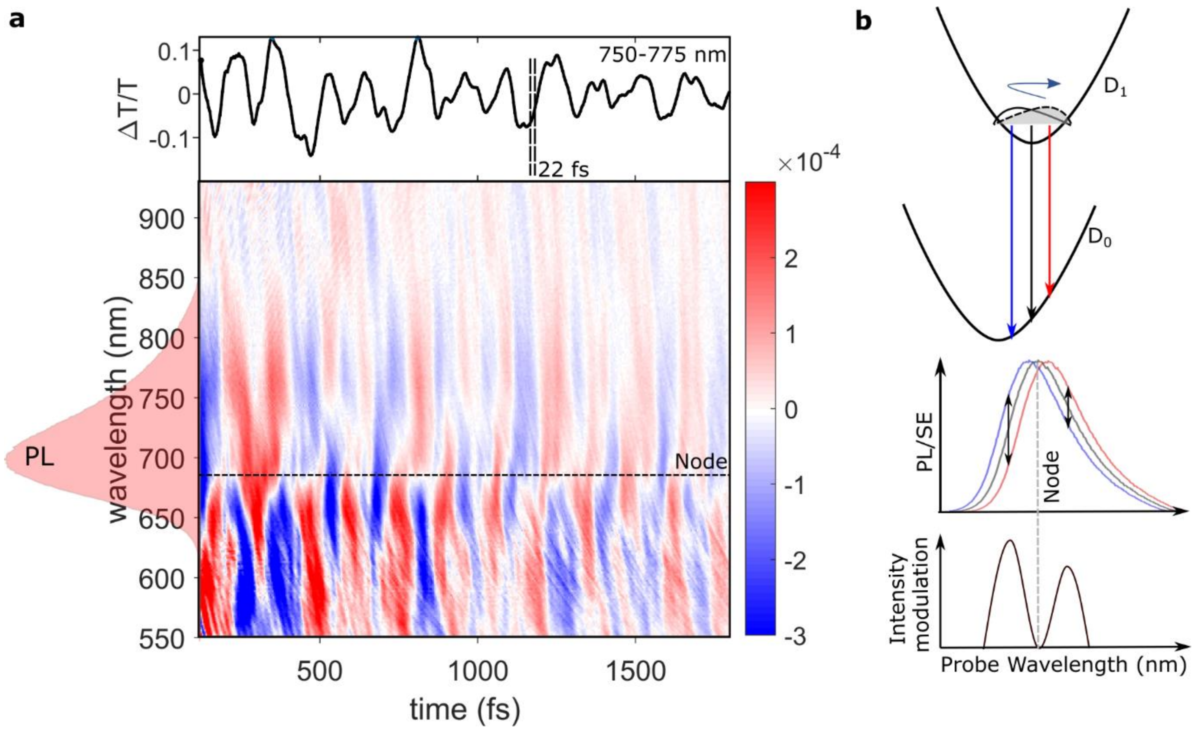

Supplementary Figure 11: **Phase Analysis and origin of the high-frequency decoupled vibrational coherence**: a, Pump-probe map (TTM-3PCz) showing coherent oscillations after fitting the population kinetics and subsequently subtracting from convoluted kinetics at each probe wavelength. The wavelength snap (750-775 nm) is plotted on the upper panel. The reference 22 fs indicates a typical oscillation period of a high frequency C=C stretching mode. b, Schematic illustration explaining the spectral position of the node.

Section 7: **Excited-state impulsive vibrational spectroscopy of 4CzIPN in 3-pulse geometry**

As the blue shifted charge-transfer absorption band of 4CzIPN (see absorption spectra in Supplementary Figure 12a) is not directly accessible by the impulsive pumps available to our system (see methods), we performed a 3-pulse actinic pump excitation followed by an impulsive 'push' pulse to generate nuclear wavepacket motion on the excited state surface[6]. The 200 fs actinic pump is spectrally tunned to 450 nm to photo-excite the ^{1}CT state[7]. The transient absorption spectra plotted in Supplementary Figure 12a reveal a negative differential transmittance signal (600-900 nm) which corresponds to the photo-induced absorption feature of the CT-type S_1 state to higher excited state. The impulsive push pulse (Supplementary Figure 12a, red) is spectrally tuned to the NIR region so that it has lower photon energy than lowest energy absorption band. The push pulse in compressed through the quartz window of the 200-micron cuvette by a pair of chirp mirror and the temporal profile of the compressed impulsive push pulse is characterised by SHG-FROG. The FWHM of the retrieved pulse is 8.2 fs (Supplementary Figure 12b). In Supplementary Figure 12c, the wavelength-resolved off-resonant IVS map of acetonitrile in 200-micron cuvette performed with the same impulsive push pulse is plotted. The observation of the C-H stretch mode at ~2900 cm^{-1} with significant

intensity demonstrates that the pump pulse in this case is well-compressed (< 11.5 fs, the period of oscillation for a mode at 2900 cm^{-1}) and is sufficient to observe modes having frequencies less than 2900 cm^{-1}. To reveal the excited state (^{1}CT) vibrational coupling, 500 fs after the initial photoexcitation with 450 nm actinic pump, the excited state population is re-excited with the impulsive push pulse. Vibrational coherence generated on the excited surface is probed by the time delayed white-light probe (same as described in the method section).

The impulsive vibrational map is generated by fast Fourier transforming the excited state coherence after excluding the ground state contribution which is plotted in the Extended Data Fig. 4b (1 mm cuvette) and Supplementary Figure 12d (200-micron cuvette). The excited state vibrational spectrum is dominated by low frequency modes (244, 428 cm^{-1}). No significant intensity is obtained from the high-frequency Raman modes. Additionally, as depicted in the Supplementary Figure 13, no significant difference in spectra is observed between the photo-induced absorption and stimulated emission-rich probe region which strongly indicates the low-frequency vibrational modes are the strongly coupled modes in the generated nuclear wavepacket on the ^{1}CT surface.

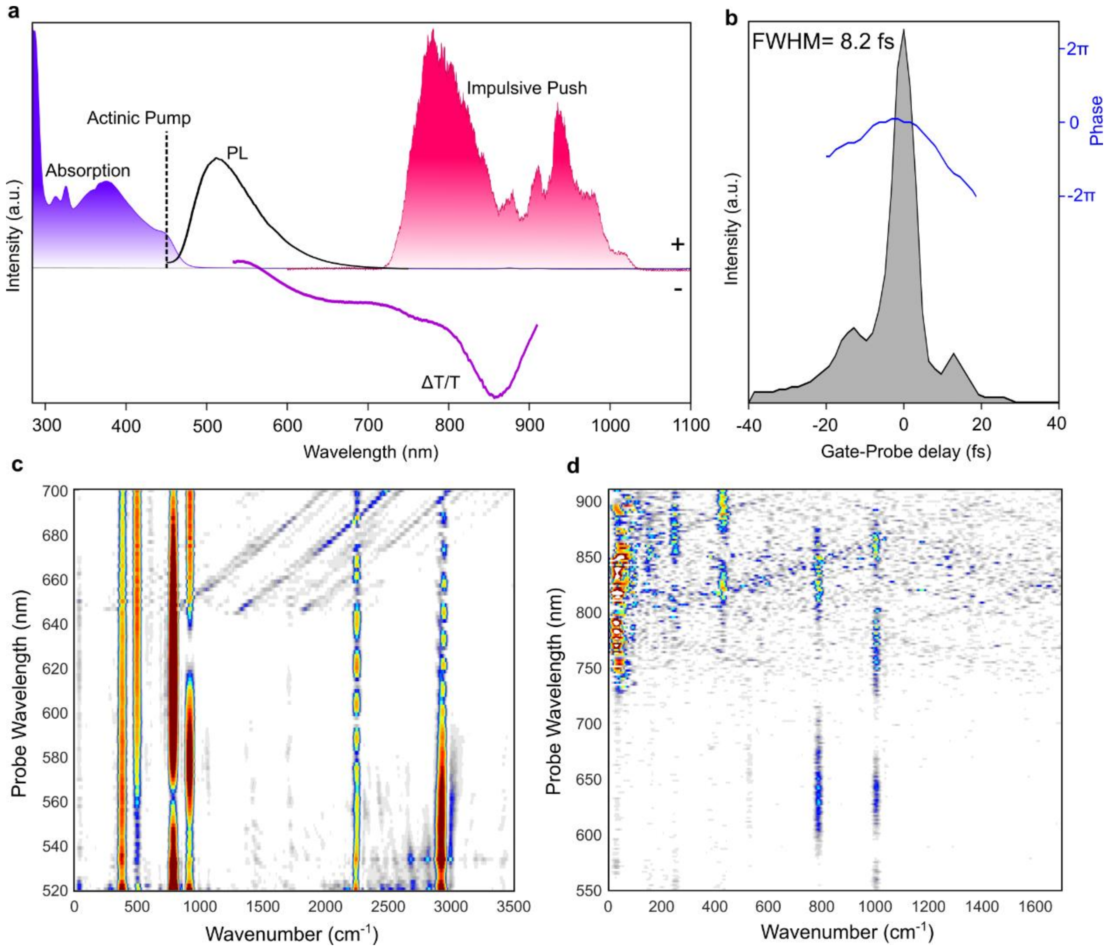

Supplementary Figure 12: **Detailed description of the 3-pulse IVS experiment performed on 4CzIPN: a,** absorption spectra of 4CzIPN solution in Toluene (blue), wavelength of actinic pump (dotted black),

photoluminescence spectra (black line), transient absorption (photoinduced absorption) signature (purple), spectra of the impulsive push (red). **b,** Temporal profile of the impulsive push pulse retrieved with SHG-FROG. **c,** off-resonant IVS map of acetonitrile showing significant intensity at 2900 cm^{-1}. d, Experimental excited-state Raman map of 4CzIPN (toluene, 200-micron quartz cuvette)

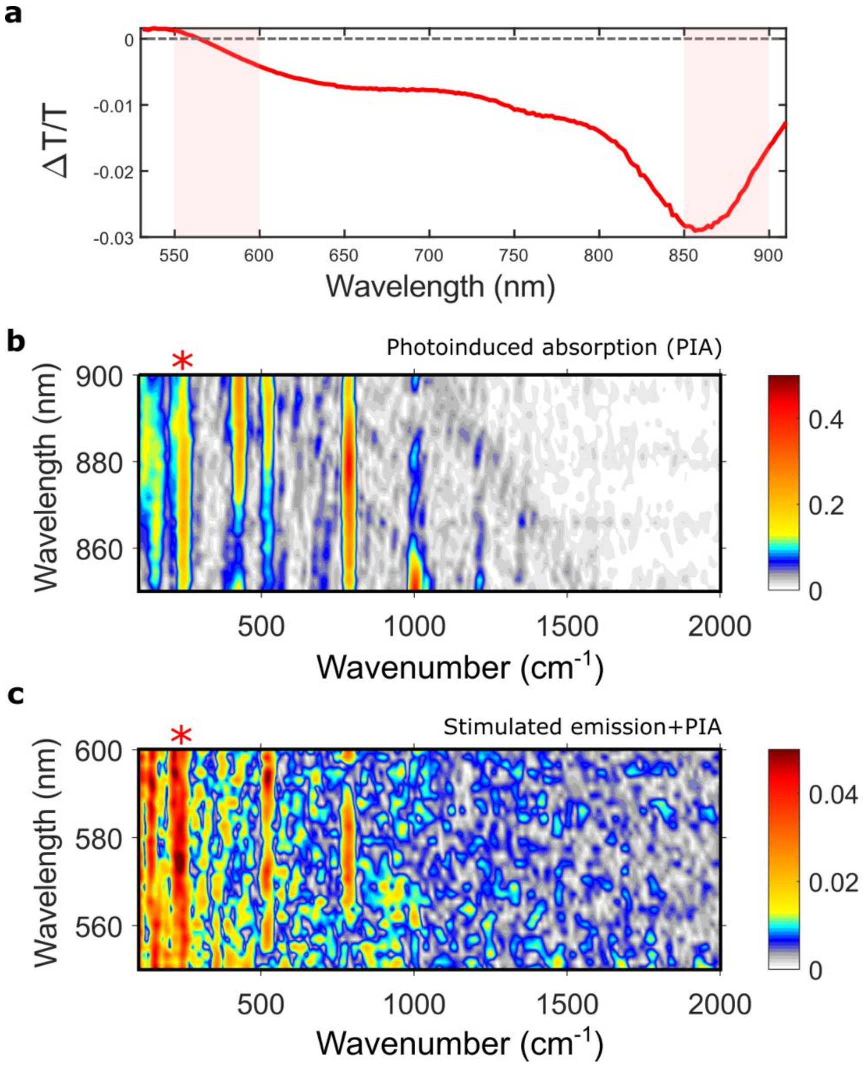

Supplementary Figure 13: **Detailed description of the probe wavelength resolved IVS spectra of 4CzIPN: a,** Transient absorption signal (at 10 ps) of 4CzIPN (toluene) upon pumping at 450 nm. **b,** IVS map (850-900 nm) extracted from the vibrational coherence generated at the PIA regime. c**,** IVS map (550-600 nm) extracted from the vibrational coherence generated at the SE+PIA regime. Asterisk indicates 244 cm^{-1} mode which one of the prominent *modes* coupled to the 4CzIPN exciton.

Section 8: **Ultrafast dynamics of TTM-TPA (Band selective excitation)**

As reported earlier[8], for this class of TTM-donor molecules, no significant difference in photoluminescence quantum yield is observed upon photoexciting to the higher lying local excited state and lower lying charge transfer state. Hence, it can be concluded that higher excited state population (D_2 exciton) rapidly internally convert to the lowest excited charge transfer state (D_1 exciton).

The ultrafast transient absorption response from TTM-TPA upon photo exciting the D_1 state (P_1 pulse) and the D_2-rich state (P_2 pulse) is shown in Extended Data Fig. 5a. The whole visible probe is dominated by the negative differential transmittance signal (photo-induced absorption) upon exciting with both pulses. At ultrafast timescale (100-200 fs), P_2 excited negative feature is red-shifted by 30 nm with respect to the P_1 excited feature. At later time scale (2 ps), the photo-excited absorption features excited by both pulses appear to be converged. As the higher-lying excited states (D_n, n≥3) are energetically very close[9], the photo induced absorption (PIA) from the D_2 state is expected to be energetically lower with respect to the PIA from the D_1 state (schematised in the Extended Data Fig. 5b, inset). That indicates the early time population, which is photo generated by the P_2 pulse, corresponds to the D_2 excited state and the internal conversion from D_2 to the D_1 is almost completed in 2 ps. To estimate the temporal dynamics of the internal conversion, we compute a wavelength dependent scaling factor at 2 ps of the ΔT/T (P_1-excited) to the ΔT/T (P_2-excited). Therefore, temporal dynamics of the parameter $\left\{\frac{\Delta T}{T}(P_2) - \frac{\Delta T}{T}_{scaled}(P_1)\right\}$, should obtain the lifetime of D_2 exciton (internal conversion timescale for $D_2 \rightarrow D_1$), which is plotted in the Extended Data Fig. 5b for a representative wavelength (790-800 nm) and turns out to be 670±125 fs.

Section 9: **Analysis of the low frequency vibrational modes of TTM-3PCz**

The experimentally obtained excited state Raman spectra of TTM-3PCz (see Extended Data Fig. 3a) depicts vibrational modes with frequency 232 cm^{-1} and 150 cm^{-1} shows the strongest exciton-vibration coupling. To visualize the nature of these two normal modes we calculate all the normal modes of TTM-3PCz and corresponding coupling to the $D_0 \rightarrow D_1$ transition (see the Huang-Rhys Factor vs mode frequency plot at Extended Data Fig. 3b). Very strongly coupled modes at 156, 202 and 204 cm-1 should correspond to experimentally obtained modes. As shown in Extended Data Fig. 3c, the mode at 150 (156) cm^{-1} corresponds to structural deformation motion which include out of plane ring bending at the TTM part and in plane ring deformation at 3PCz part. The vibrational mode at 232 (202/204) cm^{-1} is associated with a torsional mode predominantly localised in the junction of the donor(3PCz) and acceptor (TTM).

Section 10: **Charge-transfer character**

Supplementary Figure 14 visualises the frontier molecular orbitals of APDC-DTPA, ITIC and TTM-3PCz, as obtained from our DFT calculations. Our TD-DFT calculations indicate that the first excited state in these systems is predominantly HOMO to SOMO/LUMO, hence the spatial overlap of these orbitals determines the charge transfer character of the lowest excited state in these structures. Evidently, in APDC-DTPA and TTM-3PCz, there

is little spatial overlap between the two, making the charge transfer character dominant. On the other hand, in ITIC (NFA) there is large overlap between the orbitals, giving the excitation a weak charge-transfer character.

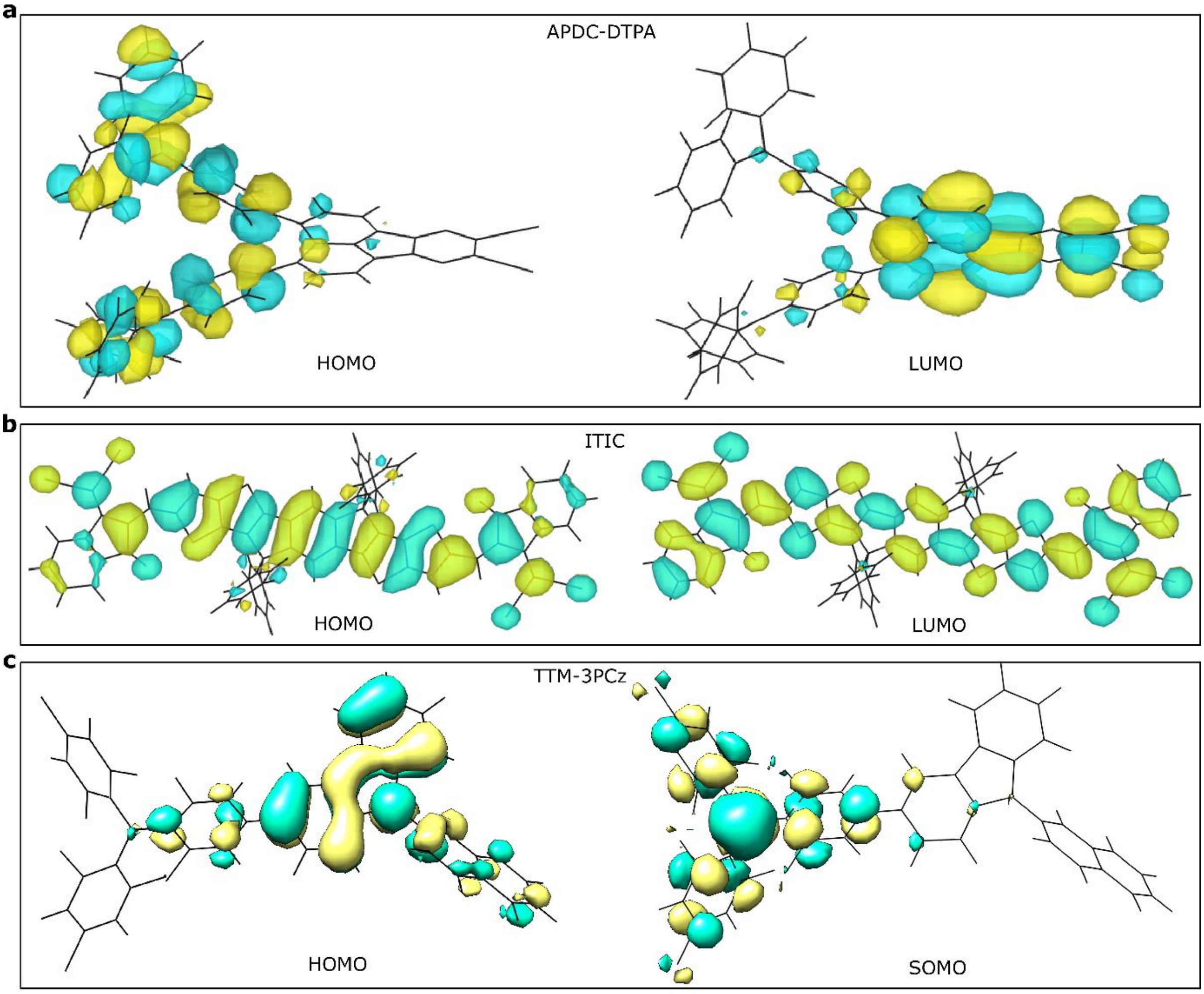

Supplementary Figure 14: HOMO and LUMO/SOMO of the representative TADF (APDC-DTPA), NFA (ITIC), radical (TTM-3PCz)

Section 11: **Electronic structure Calculation of TTM-TPA**

Unrestricted Kohn-Sham time-dependent density-functional theory (TDDFT) calculations using the B3LYP functional and cc-pVDZ basis set give good agreement with experiment for the transition energy of the lowest three absorption bands, which are listed in Extended Data Table 2. The contributions of the different molecular orbitals involved in the transitions are also given in Extended Data Table 2. This is in agreement with previous calculations[10] performed on the similar derivative TTM-3NCz, showing electronic transitions taking place from closed-shell orbitals to the singly-occupied molecular orbitals (SOMO), for the first three excited states.

Section 12: **Calculation of exciton-vibration interactions in studied molecules**

We start by describing the mode frequency-resolved exciton-vibration coupling of TTM-TPA as a model system of an efficient radical emitter. Following a geometry optimization at the ground electronic state using the cc-pVDZ basis set and B3LYP functional, we calculate the vibrational modes within the harmonic approximation and a scaling factor of 0.9688 is applied to the vibrational frequencies[11]. We analysed 172 out of 192 modes with vibrational frequencies $(100\, cm^{-1} \leq \omega(k) \leq 2000\, cm^{-1})$, as the modes with extremely low frequency (<100 cm^{-1}) are highly anharmonic in nature[12]. Likewise, Carbon-Hydrogen stretching modes with very high frequency (>2000 cm^{-1}) are known to couple to the exciton weakly[13] at the single molecule level (excluding the H-bonding in an aggregate[14]) and are also beyond our experimental scope.

For the excited electronic states i of interest we calculate the exciton-vibration coupling for each mode k as:

$$V_{ev(i)}\,(k) = \frac{E_{ex(i)}^{+\delta u_k} - E_{ex(i)}^{-\delta u_k}}{2\delta u(k)}, (i = D_0 \rightarrow D_1/D_0 \rightarrow D_2) \qquad \text{SI1}$$

Where δu_k represents a small unitless displacement of the equillibrium geometry along the vibrational mode, $k.$ $E_{ex(i)}^{+\delta u(k)}$ and $E_{ex(i)}^{-\delta u(k)}$ refer to the excitation energy for an electronic transition (i) upon displacing the equilibrium geometry by a small dimensionless quantity $(+\delta u, -\delta u)$ along the kth normal mode with frequency ω, in the harmonic limit.

Hence, for every vibrational mode, two TD-DFT calculations are required per excited state.

The Huang-Rhys factors are then obtained as

$$S_i(k) = \left(\frac{V_{ev(i)}\,(k)}{\hbar\omega(k)}\right)^2 \qquad \text{SI2}$$

The total reorganisation energy is approximated as[15,16]

$$\lambda = \sum_k 2\hbar\,\omega(k) S_i(k) \quad \text{SI3}$$

The vibrational reorganisation energy is approximated as[15,16]

$$\lambda_v = \sum_k \hbar\,\omega(k) S_i(k) \quad \text{SI4}$$

In order to quantify the contribution of high-frequency versus low-frequency modes to the overall exciton-vibration coupling, we compute the ratio of the vibrational reorganization energy (λ_v) due to high-frequency motions $(> 1000\, cm^{-1})$ to that due to low-frequency modes $(< 1000\, cm^{-1})$:

$$\varphi_{lf}^{hf} = \frac{\lambda_v^{hf}}{\lambda_v^{lf}}; \text{ where } \lambda_v^{hf} = \sum_{\omega_k=1000}^{2000} \hbar\omega_k S_{ev}(k);\ \lambda_v^{lf} = \sum_{\omega_k=100}^{1000} \hbar\omega_k S_{ev}(k) \qquad \text{SI5}$$

Extended Data Fig. 8a-b visualizes the mode-resolved contributions to the vibrational reorganization energy (λ_v) of the D_1 and D_2 states of TTM-TPA, from where it becomes evident that high-frequency modes play a significantly less prominent role for the charge-transfer-like D_1 wavefunction. Indeed, the ratio defined above has a value of 0.66 for this state, compared to 1.93 for D_2, further emphasising this important difference.

It is important to emphasise that the larger contribution of high-frequency modes to the exciton-vibration coupling of non-CT excitons compared to charge transfer states is a general effect, which does not rely on the spin configuration of the state under question. The main parameter that controls the magnitude of this interaction is the spatial overlap of electron and hole. While for TTM-TPA this was shown for the case of doublets, the same trend holds for triplet states and singlets[17]. To highlight this fact, we compute the ratio of high-frequency to low-frequency contributions to the reorganization energy of the excited states of different molecules with varying degrees of charge transfer character, namely the first excited singlet S_1 of pentacene, the doublet D_1 of the TTM radical (rather than the TTM-TPA donor-acceptor structure which hosts a charge transfer exciton as its lowest excitation), and S_1 of APDC-DTPA molecule which exhibits thermally activated delayed fluorescence[18]. We visualise the result in Extended Data Fig. 8c, from where it becomes evident that increased charge transfer character is directly correlated with decreased coupling to high-frequency motions and hence suppressed non-radiative recombination.

Section 13: **Exciton-vibrational coupling displacing along a pair of high-frequency normal modes**

To investigate the effect of a pair of high-frequency modes on the $D_0 \rightarrow D_1$ exciton of TTM-TPA, we have performed the analysis outlined in the work of Monserrat et al[19]. We have chosen the 1561 cm^{-1} normal mode as acceptor (TTM) centred high-frequency mode (Supplementary Figure 15a), the effect of which on the CT ($D_0 \rightarrow D_1$) and Non-CT ($D_0 \rightarrow D_2$) has been extensively discussed in the main text. We have also chosen the 1614 cm^{-1} mode as a donor (TPA) centred high-frequency mode involving C=C stretching mode (Supplementary Figure 15b). The equilibrium geometry is displaced along both normal modes as shown in Supplementary Figure 15c which visualises the total ground state DFT energies along those geometries. In the harmonic limit, we choose the combinations of displacements of $u = \pm 1$ (in units of the zero-point width $1/\sqrt{2\omega}$ of each mode). The 2D interpolated colour plot (Supplementary Figure 15d) visualises the absolute value of the deviation of the excitation energy ($D_0 \rightarrow D_1$) of the displaced geometry from the equilibrium geometry ($|E-E_{eq.}|$). The black circles in Supplementary Figure 15d correspond to geometries where the structure is displaced along individual modes. The red and blue circles correspond to in-phase and out-of-phase

combinations of the two modes respectively, when they are displaced simultaneously. When the modes are displaced in-phase the change in the excited state energy becomes higher ($|E-E_{eq.}|$=**0.0388**±0.0023 eV) with respect to geometries along the individual vibrational modes (**0.0194**± 0.0038 eV). The change in the excited state energy is significantly reduced (**0.0063±** 0.0026 eV) when the two modes are displaced in an out-of-phase manner.

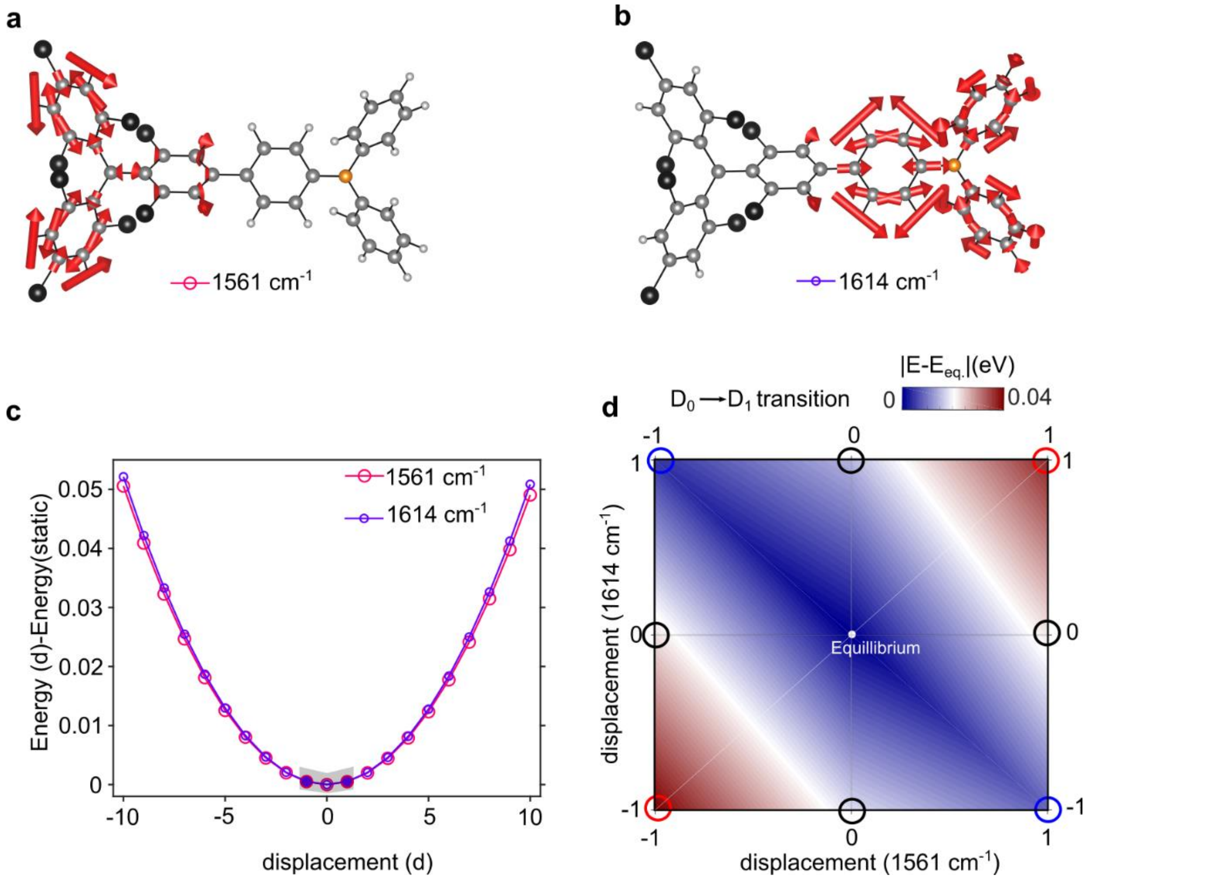

Supplementary Figure 15: **Excited state energies along a pair of normal modes: a,** vector displacement diagram of the SOMO (e^-)- centered normal mode with frequency 1561 cm^{-1}. **b,** vector displacement diagram of the HOMO (h^+)- centered normal mode with frequency 1614 cm^{-1}. **c,** ground state potential energy surface of the two normal modes. **d,** Interpolated contour plot of the differential transition energy ($D_0 \rightarrow D_1$) against displacement along the coordinate of 1561 cm^{-1} and 1614 cm^{-1} modes. The black circles represent the geometries corresponding to the displacement from the equilibrium geometry along the individual normal modes. The blue and red circles represent the geometry when the molecule is displaced along both normal modes. The in-phase and out-of-phase displacements are indicated by red and blue circles respectively.

Section 14: **Molecular orbital (MO)-vibrational coupling**

To gain insights about how individual molecular orbitals are coupled to the vibrational modes, we calculated the Huang-Rhys factor which is defined for k^{th} normal mode as -

$$S(k) = \left(\frac{E_{mo}^{+\delta u_k} - E_{mo}^{-\delta u_k}}{2\delta u(k).\hbar\omega(k)}\right)^2 \quad \text{SI6}$$

Where δu_k represents a small unitless displacement of the equillibrium geometry along the vibrational mode, $k. E_{mo}^{+\delta u_k}$ and $E_{mo}^{-\delta u_k}$ refer to the energy of a particular molecular orbital upon displacing the equilibrium geometry by a small dimensionless quantity $(+\delta u, -\delta u)$ along the kth normal mode with frequency ω, in the harmonic limit. Hence, for every vibrational mode, two DFT calculations are required per molecular orbital. Due to the highly anharmonic nature of the modes having frequency ≤100 cm^{-1}, we focus on the normal modes in the frequency range of 100-2000 cm^{-1}.

14.1: Electronic Localisation of the HOMO of non-planar donor moieties

In Supplementary Figure 16-17, we visualise the Huang-Rhys factor for the frontier molecular orbitals of the triphenylamine (TPA) and N-phenylcarbazole (PCz) which are representative of the donor moieties of the studied radical systems (TTM-3PCz, TTM-3NCz, TTM-TPA) and TADF systems (APDC-DTPA, 4CzIPN).

14.1.1: triphenylamine (TPA): As displayed in Supplementary Figure 16, for triphenylamine (TPA), the Huang-Rhys factor of the high-frequency modes (>1000 cm^{-1}) is significantly supressed for the HOMO with respect to the other frontier MOs (HOMO-2, HOMO-1, LUMO, LUMO+1, LUMO+2). For example, the C=C (Carbon double bond) stretching motion in the phenyl rings at 1611 cm^{-1} (highlighted in red circle in Supplementary Figure 16) has a Huang-Rhys factor for the HOMO that is 1/9th of the LUMO. The HOMO orbital has nitrogen p_z non-bonding character. This non-bonding type HOMO of TPA is mostly localised on the N atom and has a very weak π-delocalisation over the phenyl rings due to the non-parallel arrangement of the π-orbitals of the phenyl rings with respect to the central N-atom. Hence, this non-bonding type HOMO which is localised on the N-orbital, will be weakly perturbed upon displacing the equilibrium geometry of the TPA along the high-frequency phenylic π-stretching modes, unlike other MOs which have higher orbital coefficients on the phenyl rings in the vicinity of the C=C bonds.

14.1.2: N-phenylcarbazole (PCz): A similar result has been found for the N-phenylcarbazole (PCz) which is the donor moiety of TTM-3PCz. As visualised in Supplementary Figure 17, the HOMO possesses supressed coupling to the high-frequency modes with respect to the other molecular orbitals. Similar to TPA, the HOMO of PCz has nitrogen p_z non-bonding like character. The localisation of the non-bonding type HOMO on the central N atom is not as pronounced as TPA for steric reason. Other MOs, for instance, the LUMO, are highly delocalised over the carbon-rings. This localisation of the non-bonding type HOMO on the N-atom contributes to its reduced coupling to high frequency π-ring stretching modes with respect to the other MOs.

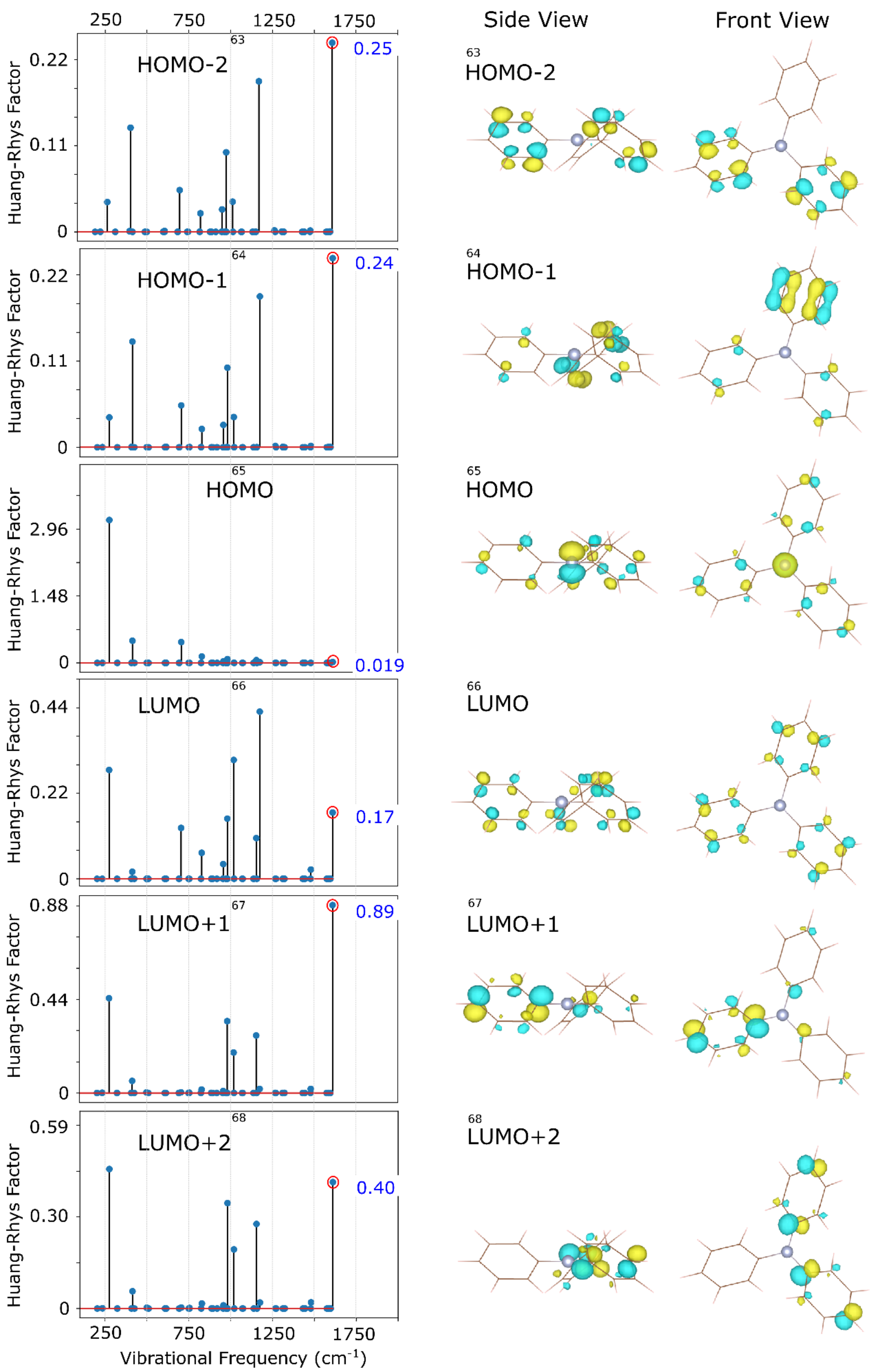

Supplementary Figure 16: **Huang-Rhys Factor of the frontier molecular orbitals of triphenylamine (TPA).** The red circle indicates the 1611 cm^{-1} mode and the respective Huang-Rhys Factor for each MO. Frontier molecular are plotted with iso-value 0.075 in the right panel of the figure, at the ground state optimised geometry.

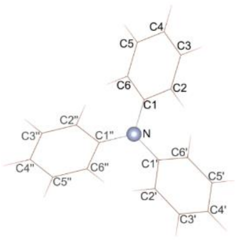

Supplementary Figure 16.2: **atom-by-atom description of TPA**

Supplementary Table 1: Nature of the molecular orbital of TPA (with iso-surface value 0.075)

MO	Assignment of the MO	Occupation	Connections	Connectivity
**63**	HOMO-2	2	C5’- C6’	Bonding
			C2’-C3’	Bonding
			C5’’- C6’’	Bonding
			C2’’-C3’’	Bonding
**64**	HOMO-1	2	C5- C6	Bonding
			C2-C3	Bonding
**65**	HOMO	2	N-C1-C2	Non-bonding
			N-C1-C6	Non-bonding
			C2-C3-C4	Non-bonding
			C4-C5-C6	Non-bonding
			N-C1’-C2’	Non-bonding
			N-C1’-C6’	Non-bonding
			C2’-C3’-C4’	Non-bonding
			C4’-C5’-C6’	Non-bonding
			N-C1’’-C2’’	Non-bonding
			N-C1’’-C6’’	Non-bonding
			C2’’-C3’’-C4’’	Non-bonding
			C4’’-C5’’-C6’’	Non-bonding
**66**	LUMO	0	C5- C6	Anti-bonding
			C2-C3	Anti-bonding
			C5’- C6’	Anti-bonding
			C2’-C3’	Anti-bonding
			C5’’- C6’’	Anti-bonding
			C2’’-C3’’	Anti-bonding
			C2-C1-C6	Non-bonding
			C3-C4-C5	Non-bonding
			C2’-C1’-C6’	Non-bonding
			C3’-C4’-C5’	Non-bonding
			C2’’-C1’’-C6’’	Non-bonding
			C3’’-C4’’-C5’’	Non-bonding
**67**	LUMO+1	0	C1’’-C6’’	Anti-bonding
			C1’’-C2’’	Anti-bonding
**68**	LUMO+2	0	C1-C6	Anti-bonding
			C1-C2	Anti-bonding
			C1’-C6’	Anti-bonding
			C1’-C2’	Anti-bonding

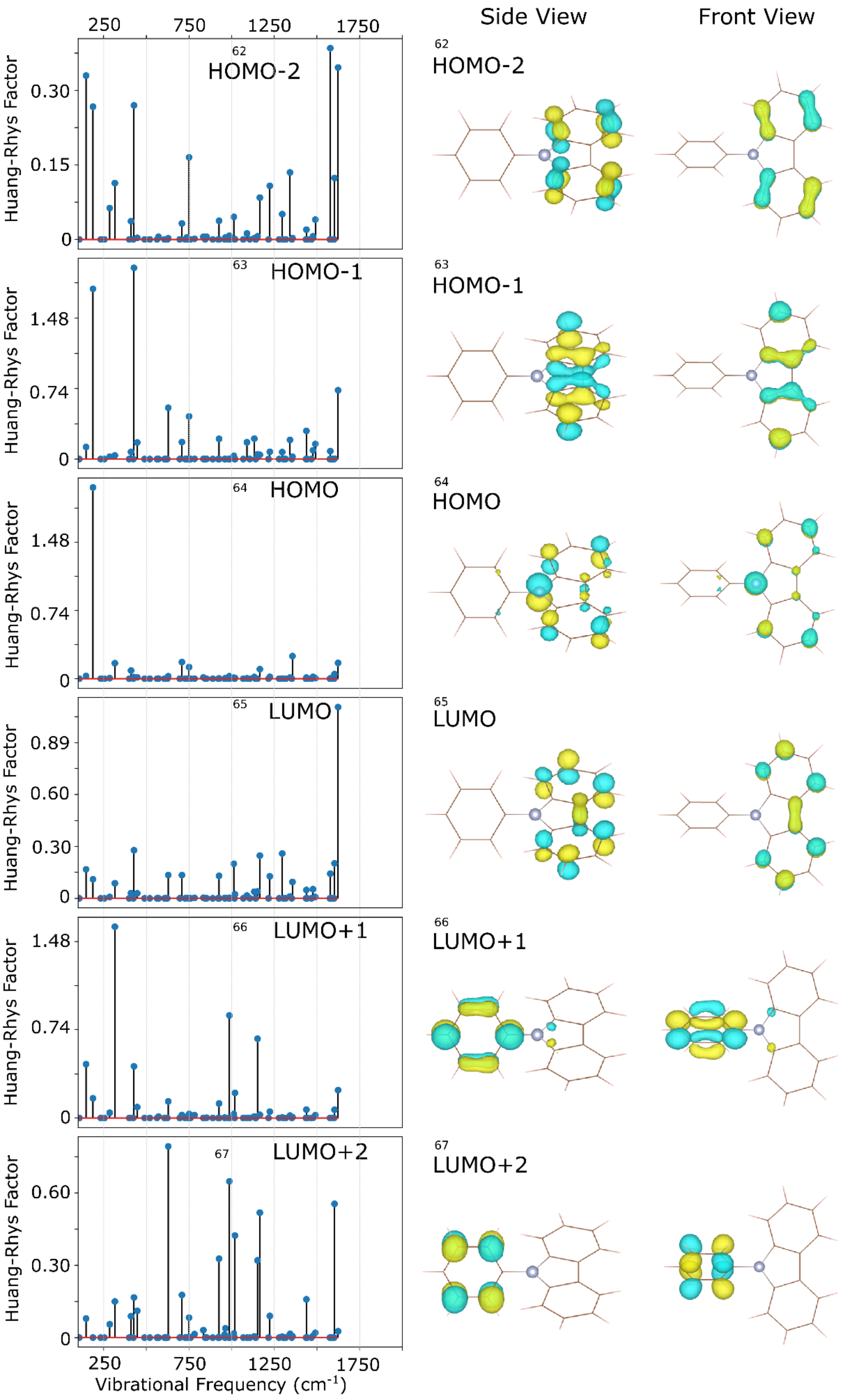

Supplementary Figure 17: **Huang-Rhys Factor of the frontier molecular orbitals of the N-phenylcarbazole (PCz)** Frontier molecular are plotted with iso-value 0.075 in the right panel of the figure at the ground state optimised geometry

14.2: Electronic Localization of the HOMO of APDC-DTPA (TADF) and TTM-TPA (radical)

In the previous subsection (14.1), we discussed how the HOMO of donor moieties (TPA, PCz) is decoupled from the high-frequency π-ring stretching vibrations due to the electronic localisation of the non-bonding type molecular orbital on the central N atom, which is more pronounced for the TPA. We have also evaluated the Huang-Rhys Factor for the frontier MOs of TTM-TPA (Supplementary Figure 18) and APDC-DTPA (Supplementary Figure 21). Both molecules have TPA as a donor moiety, where the non-bonding type HOMO localises. As expected from the results discussed in the previous subsection, the HOMO of both APDC-DTPA and TTM-TPA shows reduced coupling to high-frequency modes.

14.3: Electronic localisation of the SOMO orbital of TTM-Donor radicals

It is intriguing to notice that the SOMO in TTM-TPA shows reduced coupling compared to unoccupied MOs (LUMO, LUMO+1) for the high frequency modes. For example, along the 1572 cm^{-1} normal mode, which is the C=C stretch at one of the phenyl rings on the TTM moiety (see Supplementary Figure 18, bottom right panel), the Huang-Rhys Factor of the SOMO is $1/60^{th}$ of the HOMO. A potential explanation of the supressed coupling of the high-frequency ring stretching modes (C=C) to the orbital SOMO with respect to the LUMO and LUMO+1, is the non-bonding nature of the SOMO. As pointed out earlier[10,20] TTM-donor based radicals' non-bonding type SOMO has a very dominant orbital contribution on the central sp^2-hybridised carbon atom (see the scheme Supplementary Figure 18, right bottom panel). As a result, it is mostly localised on the central Carbon atom and it has very weak orbital coefficients in the vicinity of the phenylic pi-bonds (See Supplementary Figure 19). Hence, the SOMO energy is less perturbed in comparison the other orbitals (LUMO, LUMO+1), upon displacing the molecular geometry along the ring stretching mode (1572 cm^{-1}).

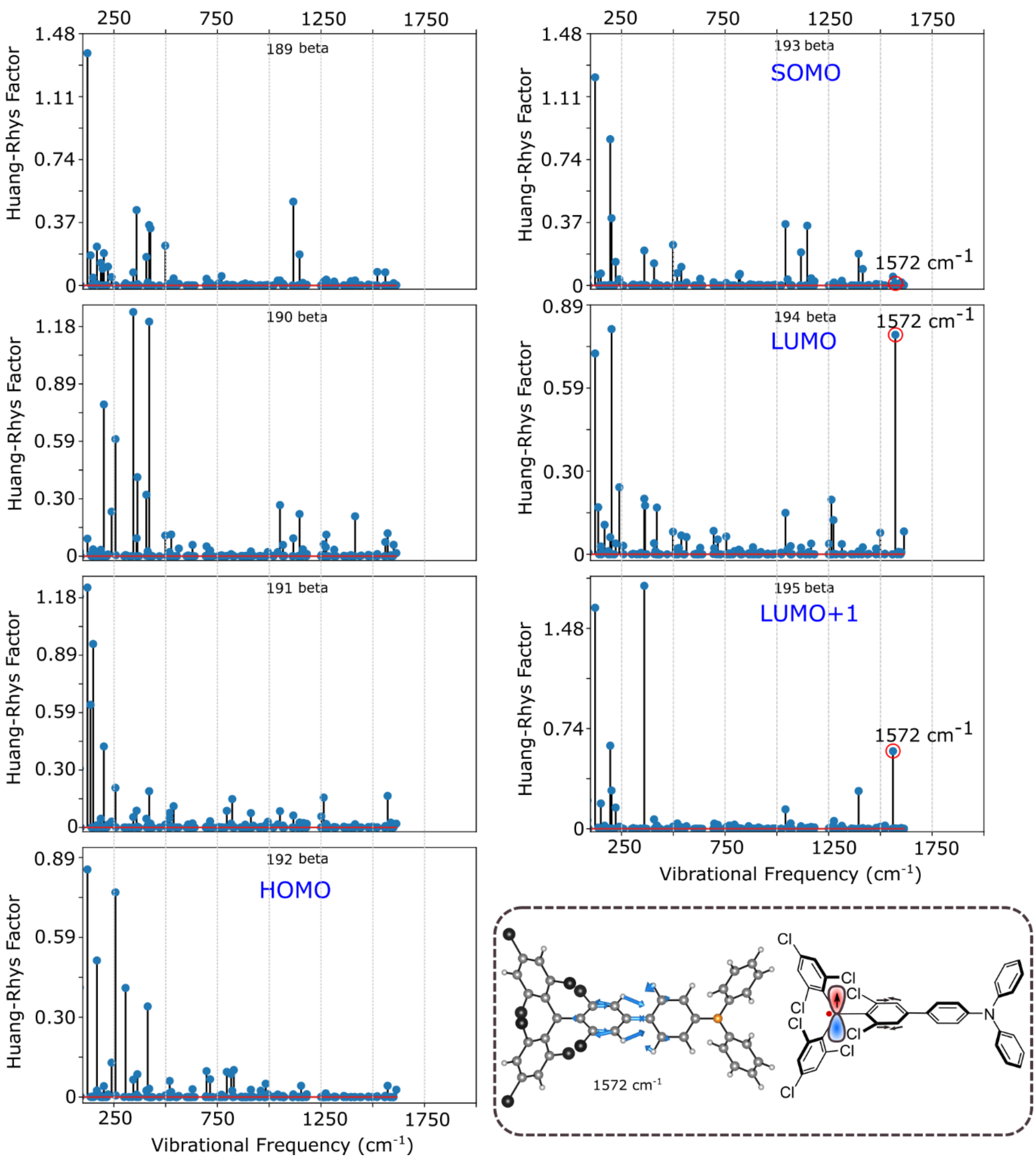

Supplementary Figure 18: **Huang-Rhys Factor of the frontier molecular orbitals of the TTMTPA.** The red circle highlights the 1572 cm^{-1} mode. The vector displacement diagram of that mode is plotted in the right bottom panel.

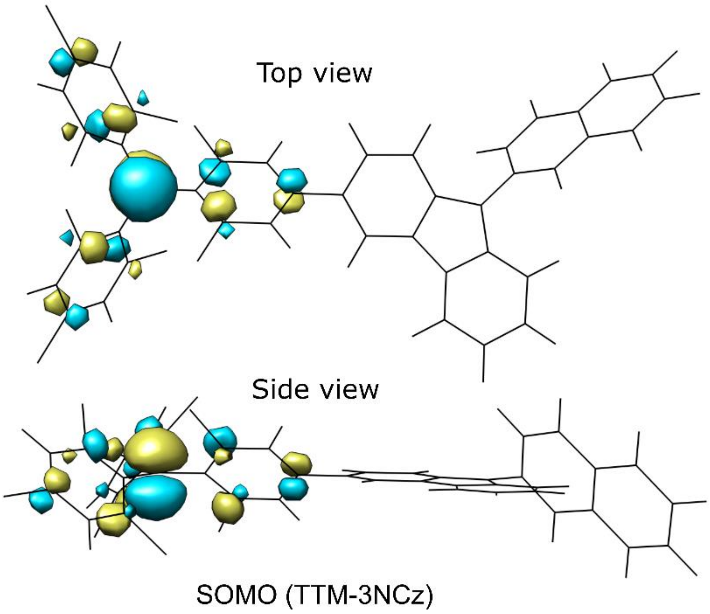

Supplementary Figure 19: **SOMO of the TTM-donor radical (representative TTM-3NCz radical adapted from Ai *et al*[10]) (**with iso-value 0.075**)**

14.4: Electronic localization and vibrational decoupling

In TTM-Donor radicals (TTM-3PCz, TTM-3NCz, TTM-TPA), the charge transfer electronic transition takes place from the donor moiety (TPA, PCz)-centred HOMO to the TTM-centred SOMO as discussed in the main text. Our first-principles calculations show that both of these frontier molecular orbitals are individually weakly-coupled to C=C stretching modes on the phenyl rings, which can be explained by these non-bonding type molecular orbital's electronic localisation on a single C or N atom.

This is further supported by visualising the exciton wavefunction (transition density) plotted in Supplementary Figure 20. For the charge transfer $D_0 \rightarrow D_1$ state in TTM-3PCz and TTM-TPA, the wavefunction is mostly localised on the sp^2 hybridised central carbon and it has a reasonable contribution on the N atom's p-orbital in the donor moiety which does not overlap with the phenylic C=C bonds. On the contrary, the exciton wavefunction/transition density for the $D_0 \rightarrow D_2$ transition (HOMO-2 → SOMO: 80%) in TTM-TPA is spread over the phenyl rings of the TTM moiety as the lower lying occupied orbitals have π-character unlike the non-bonding p-orbital-centred SOMO[10]. Hence, the vibrational coupling in the high frequency regime (C=C stretch) for the $D_0 \rightarrow D_2$ transition is higher than the $D_0 \rightarrow D_1$ transition, which is consistent with the experimental results obtained for the TTM-TPA radical (Fig. 3, main text).

For the TADF molecule APDC-DTPA, the TDDFT results suggest that the lowest energy charge transfer transition takes place from the TPA-centred HOMO to the ACDC-centred LUMO (98.6%). As predicted from the vibrational coupling of the TPA HOMO (Supplementary

Figure 16), the HOMO of APDC-DTPA, which is TPA-rich, shows suppressed coupling to high-frequency modes compared to other frontier MOs (see Supplementary Figure 21). The LUMO of APDC-DTPA, is mostly located on the acenaphthylene based acceptor APDC (see Supplementary Figure 21) and in the vicinity of the π-bonds, hence shows reasonably strong coupling to the high-frequency modes.

Overall, the hypothesis of individually high frequency vibrations decoupled from the HOMO and the LUMO/SOMO (see Supplementary Figure 22) clearly explains the results obtained for the TTM-donor type radicals (presented in Fig 2-3, main text). This hypothesis also explains part of the results obtained for the TADF molecules (APDC-DTPA, 4CzIPN), as the planar acceptor-moiety-rich LUMO still shows reasonable coupling to the high-frequency modes.

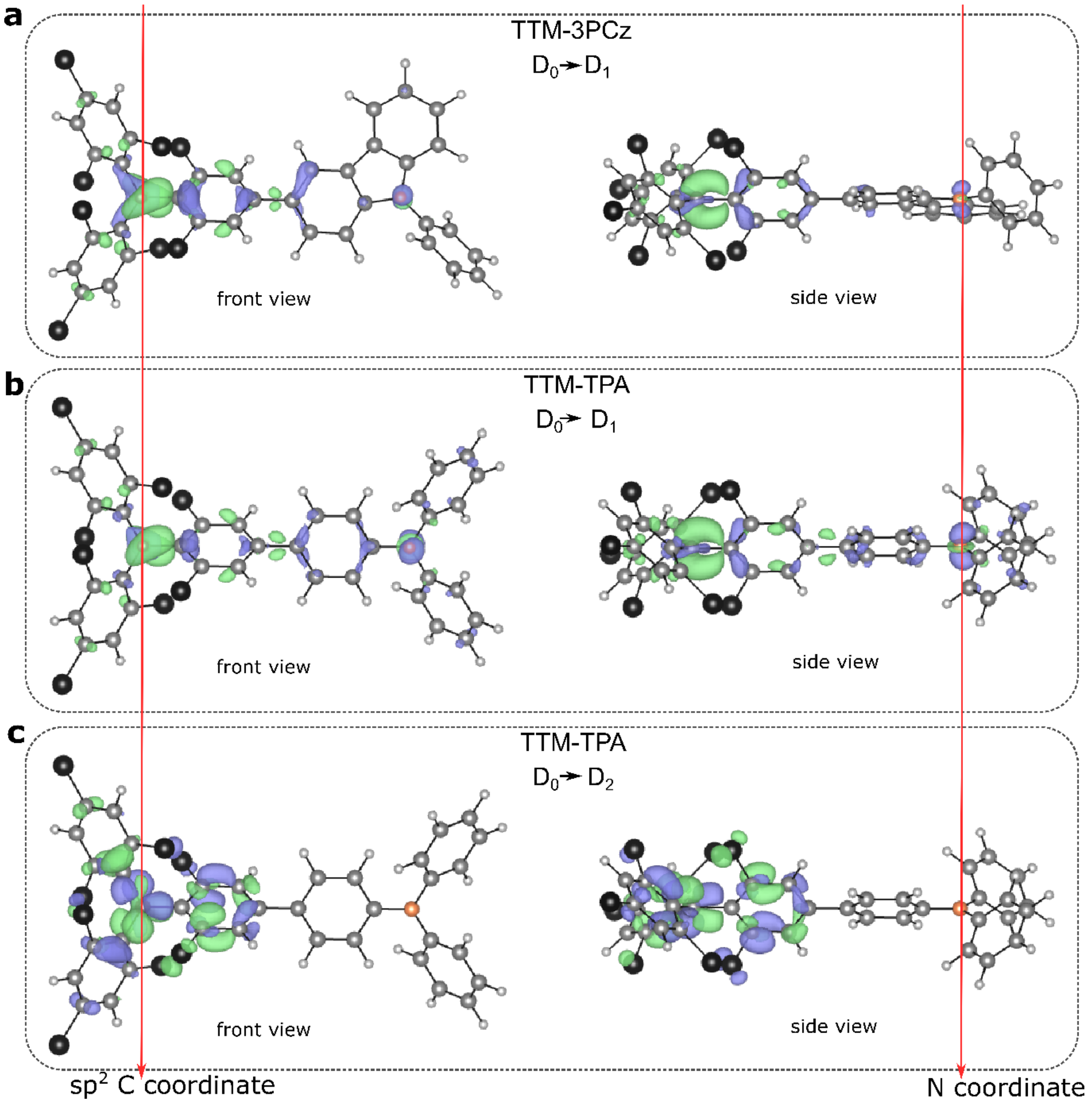

Supplementary Figure 20: **High-resolution (higher isosurface value) exciton wavefunction (transition density) plots of the $D_0 \rightarrow D_1$ transition in TTM-3PCz, $D_0 \rightarrow D_1$ transition in TTM-TPA, $D_0 \rightarrow D_2$ transition in TTM-TPA** The red arrows indicate the atomic coordinate of the sp^2 hybridised Carbon (radical centre in TTM moiety) and N-atom in the donor moiety. (plotted with iso-value 0.075)

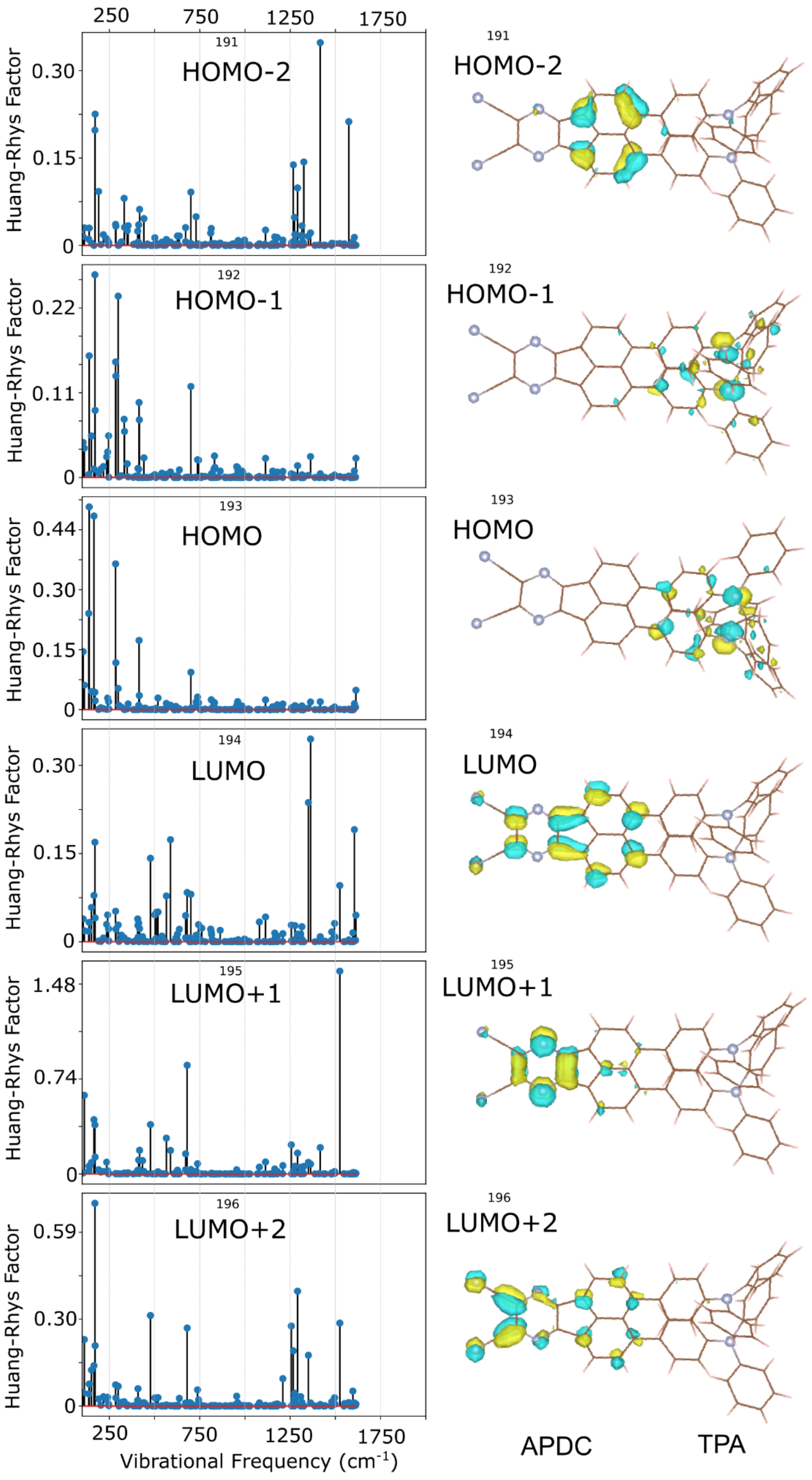

Supplementary Figure 21: **Huang-Rhys Factor of the frontier molecular orbitals of the TADF studied APDC-DTPA (with iso-value 0.075).**

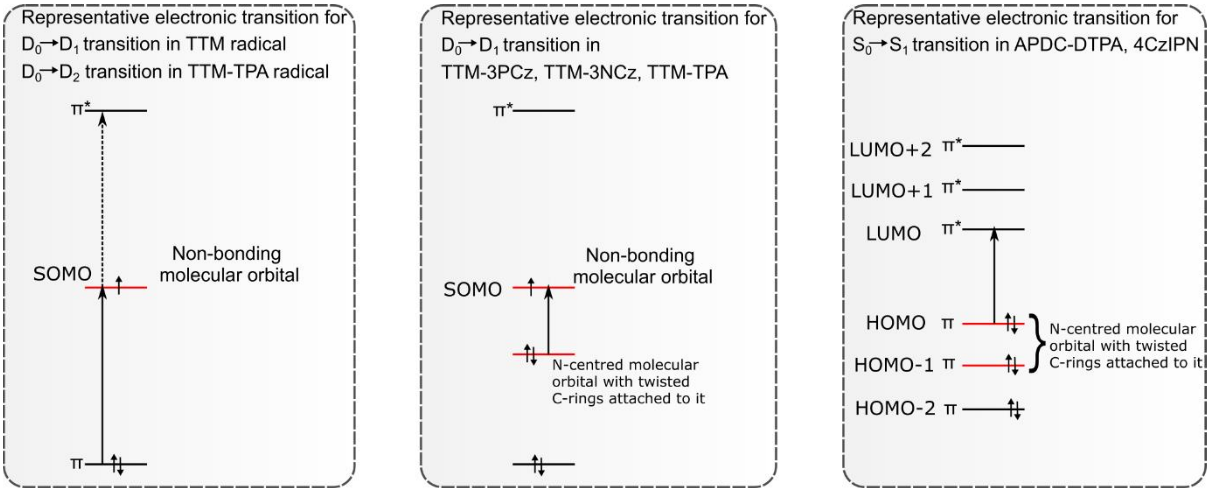

Supplementary Figure 22: **Hypothesis of individually high-frequency decoupled HOMO and LUMO/SOMO:** The MOs which are highlighted with red, have supressed high-frequency coupling revealed by first principles calculations

Section 15: **Zero-point renormalisation of excited state energies using Monte Carlo sampling**

Following the procedure outlined in detail in Hele *et al.*[21], we compute the vibrationally-renormalised energies for the D_1 and D_2 states of TTM-TPA. Briefly, the excited state energy within the harmonic approximation is:

$$E_{exc}(T) = \int d\boldsymbol{u}|\Phi(\boldsymbol{u};T)|^2 E_{exc}(\boldsymbol{u}) \text{ SI7}$$

where we integrate over the atomic configuration $\boldsymbol{u}$ and $|\Phi(\boldsymbol{u};T)|^2$ is the harmonic density distribution:

$$|\Phi(\boldsymbol{u};T)|^2 = \prod_\nu (2\pi\sigma_\nu^2(T))^{-1/2} \exp\left(-\frac{u_\nu^2}{2\sigma_\nu^2(T)}\right) \text{ SI8}$$

with every Gaussian having a temperature-dependent width:

$$\sigma_\nu^2(T) = \frac{1}{2\omega_\nu} \cdot \coth\left(\frac{\omega_\nu}{2k_B T}\right) \text{ SI9}$$

We perform a Monte Carlo sampling of the integral of equation SI7, by generating 100 atomic configurations $\boldsymbol{u}$ distributed according to $|\Phi(\boldsymbol{u};T)|^2$, and hence compute the vibrationally-renormalised excited state energy as the simple average:

$$E_{exc}(T) = \frac{1}{N}\sum_i E_{exc}(\boldsymbol{u}_i) \text{ SI10}$$

where $N = 100$ in our case. At every configuration, the excited state energy is computed within TD-DFT, using the same computational setup as that outlined in Section 12 of the SI. We find

that 100 configurations are well above the number required to reach convergence, as also shown in Supplementary Figure 23 for the example of the D_2 state of TTM-TPA, where we plot the vibrational renormalisation as a function of the atomic configuration of the molecule, also showing the running average in blue. Evidently 20-30 configurations are already sufficient for convergence, and similar results are found for all excited states studied here.

For the D_1 state, the vibrational renormalisation of its energy at 0 K due to molecular vibrations (*i.e.* the zero-point renormalisation – ZPR) is found to be $ZPR(D_1) = (-17 \pm 8)$ meV, while for the D_2 state this is equal to $ZPR(D_2) = (-65 \pm 9)$ meV. At 0 K vibrational modes are only active with their zero-point energy $\hbar\omega/2$, making the effect of high-frequency modes more prevalent. For this reason, we find that the D_2 state is renormalised by a greater amount than the CT-like D_1 state, since its spatially-localised character dictates a stronger coupling to high-frequency intra-molecular motions. This further supports the mode-resolved picture discussed in the main manuscript, wherein CT-like excitons predominantly couple to low-frequency motions, whereas spatial localisation of excited states favours coupling to high-frequency motions.

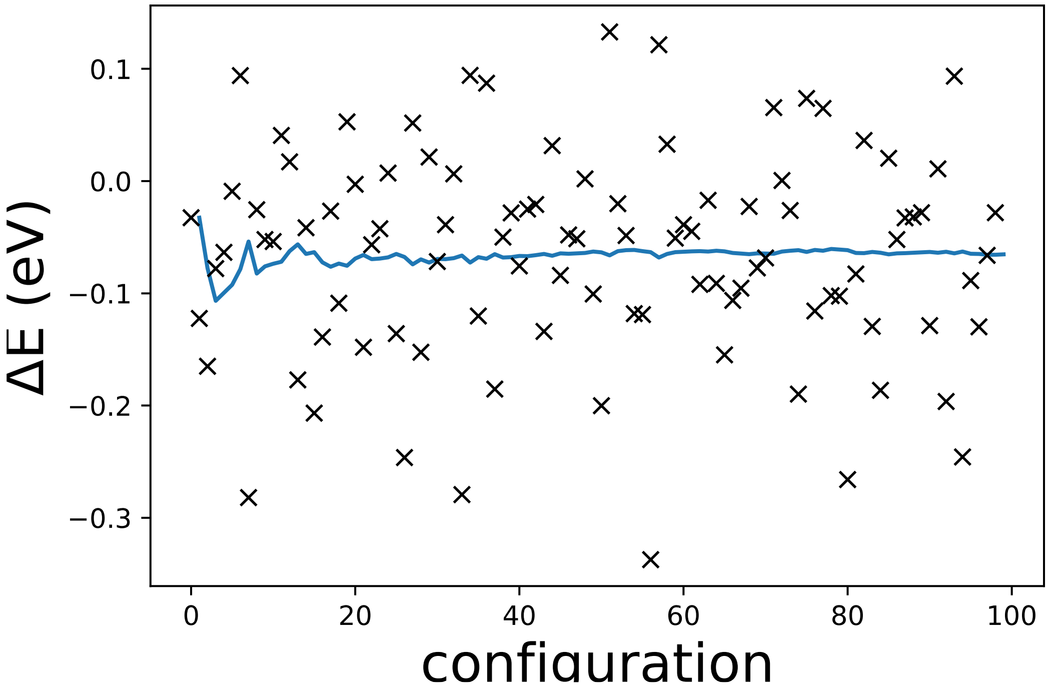

Supplementary Figure 23: **Convergence of Monte Carlo sampling of the D_2 excited state energy of TTM-TPA.**

Section 16: **Summary of the efficient red LEDs**

Supplementary Table 2: Summary for maximum EQEs and peak wavelengths of different kinds of red LEDs with EL peak wavelength >650nm adapted from Ai *et al*[10] (Purely organic emitting systems are highlighted in red).

LED	Emitting component		EL (nm)	EQE_{max} (%)
**Radical LEDs**	Spin radical based organic semiconducting molecule	TTM-3NCz[10]	710	26.5
		TTM-3PCz[10]	703	16.6
**TADF LEDs**	Thermally activated delayed fluorescence based organic molecule	APDC-DTPA[22]	693	10.19
		TPA-QCN[23]	656	12.8
**QLEDs**	Quantum Dots	CdSe/CdTe[24]	705	6.19
**PLEDS**	Perovskites	[25]	763	11.7
**PhosLEDs**	Phosphorescent metal-organic MLCT systems	Pt-based[26]	740	24±1
**Fluorescent LED**	Organic fluorophore molecule	TPANSeD[27]	664	3.8

Section 17: **Synthesis of M2TTM-3PCz and M2TTM-2PCz**

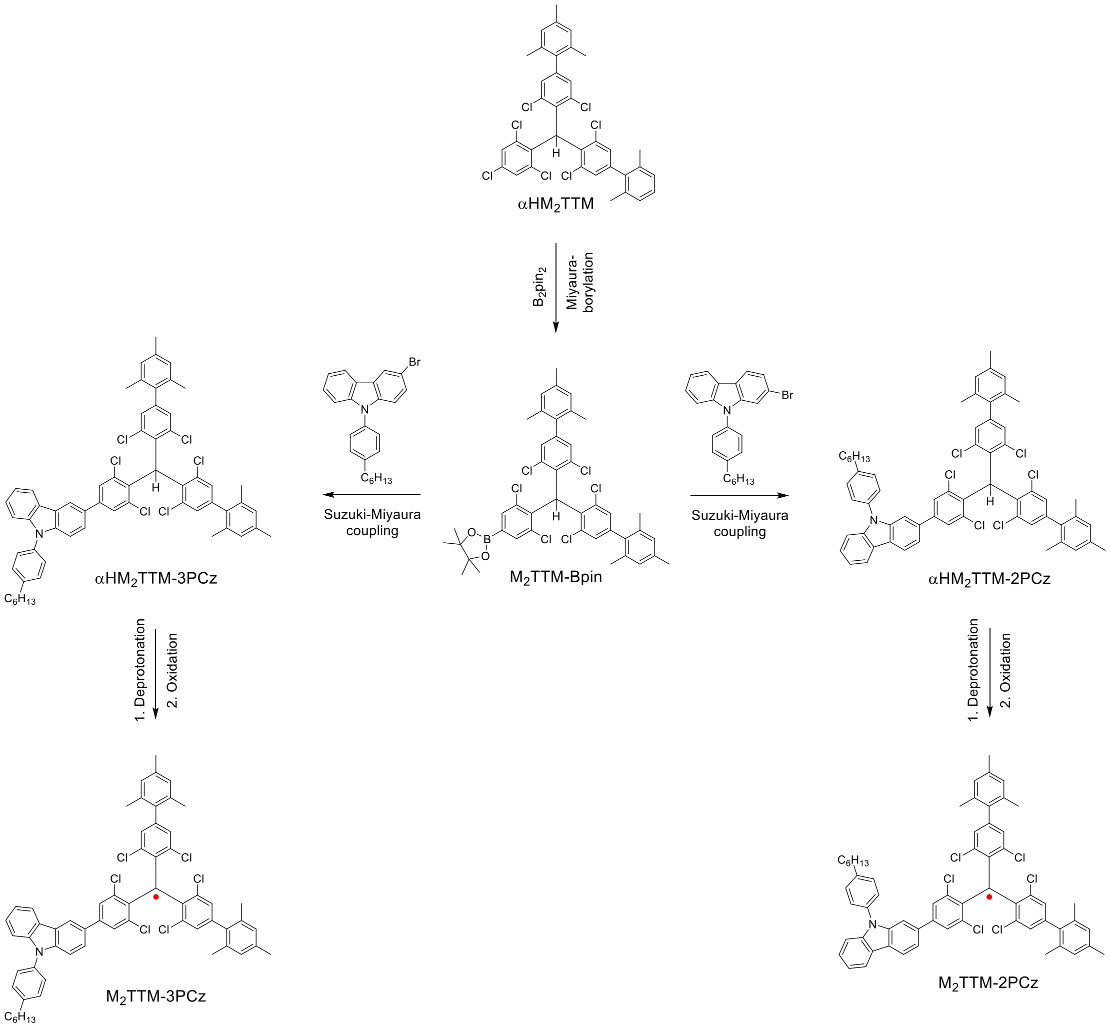

Supplementary Figure 24: Schematic synthetic pathways for M_2TTM-3PCz and M_2TTM-2PCz radicals.

Characterization and techniques

NMR spectra were recorded on Bruker Avance 400 MHz (^{1}H, 400 MHz; ^{13}C, 100 MHz) spectrometer. Chemical shifts are reported in δ (ppm) relative to the solvent peak: chloroform-*d* ($CDCl_3$: ^{1}H, 7.26 ppm; ^{13}C, 77.16 ppm). For NMR monitored reactions the spectra were referenced against 1,3,5-trimethoxybenzene (^{1}H, 6.09 ppm) as the internal standard. Mass spectra were obtained using a Waters Xevo G2-S benchtop QTOF mass spectrometer (equipped with an atmospheric solids analysis probe, ASAP) by the mass spectrometry service at Yusuf Hamied Department of Chemistry, University of Cambridge. C, H, N combustion elemental analyses (EA) were obtained on an Exeter Analytical Inc. CE-440 elemental analyser and the results are reported as an average of two samples. Flash chromatography was carried out using Biotage® Isolera™ Four System and Biotage® SNAP/Sfär Silica flash cartridges.

Materials and synthesis

Synthetic pathways for M_2TTM-3PCz and M_2TTM-2PCz are shown in Fig. 1. Preparation of 4',4'''-((2,4,6-trichlorophenyl)methylene)bis(3',5'-dichloro-2,4,6-trimethyl-1,1'-biphenyl) (αHM_2TTM), 2-(4-(bis(3,5-dichloro-2',4',6'-trimethyl-[1,1'-biphenyl]-4-yl)methyl)-3,5-dichlorophenyl)-4,4,5,5-tetramethyl-1,3,2-dioxaborolane (M_2TTM-Bpin) and 3-bromo-9-(4-hexylphenyl)-9*H*-carbazole and M_2TTM-3PCz are described in our previous publication.

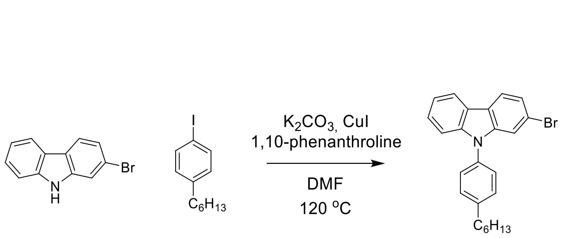

2-Bromo-9-(4-hexylphenyl)-9*H*-carbazole. Following modified literature procedure,[1] 2-bromo-9*H*-carbazole (2.000 g, 8.13 mmol, 1 equiv.), CuI (0.232 g, 1.22 mmol, 0.15 equiv.), 1,10-phenanthroline (0.220 g, 1.22 mmol, 0.15 equiv.) and K_2CO_3 (2.246 g, 16.25 mmol, 2

equiv.) were added into an oven dried 50 mL two-neck round-bottom flask. The solids were dried under vacuum for 1 h and the flask was subjected to three vacuum/Ar gas refill cycles. Anhydrous DMF (35 mL) and 1-hexyl-4-iodobenzene (3.513 g, 12.19 mmol, 1.5 equiv.) were added and the mixture was bubbled with Ar gas for 30 min, then refluxed at 120 °C for 22 h. After cooling to RT, the mixture was diluted with hexane and poured over water. The aqueous phase was acidified with 10% HCl solution. The organic phase was extracted with water (5 × 200 mL) and then dried over anhydrous $MgSO_4$. Solvent was removed under vacuum and the crude product was purified with column chromatography over silica gel, using hexane as the eluent. After removing the solvent in vacuum, the target compound was collected as colorless oil which slowly solidified to give white solid (2.934 g, 89%). ^{1}H NMR (400 MHz, $CDCl_3$) δ 8.10 (d, J = 7.8 Hz, 1H), 7.98 (d, J = 8.2 Hz, 1H), 7.52 (d, J = 1.8 Hz, 1H), 7.44–7.34 (m, 7H), 7.29 (td, J = 7.5, 1.2 Hz, 1H), 2.74 (t, J = 7.8 Hz, 2H), 1.79–1.67 (m, 2H), 1.49–1.33 (m, 6H), 0.94 (t, J = 7.1 Hz, 3H). ^{13}C NMR (100 MHz, $CDCl_3$) δ 1 143.08, 142.01, 141.42, 134.60, 130.09, 127.06, 126.41, 123.02, 122.77, 122.31, 121.55, 120.37, 120.35, 119.59, 113.00, 110.19, 35.87, 31.89, 31.57, 29.23, 22.78, 14.27. TOF-MS-ASAP$^+$ Calcd. for $[C_{24}H_{25}BrN]^+$: 406.1170. Found: m/z = 406.1165. EA Calcd. for $C_{24}H_{24}BrN$: C, 70.94; H, 5.95; Br, 19.66; N, 3.45. Found: C, 70.24; H, 5.88; N, 3.55.

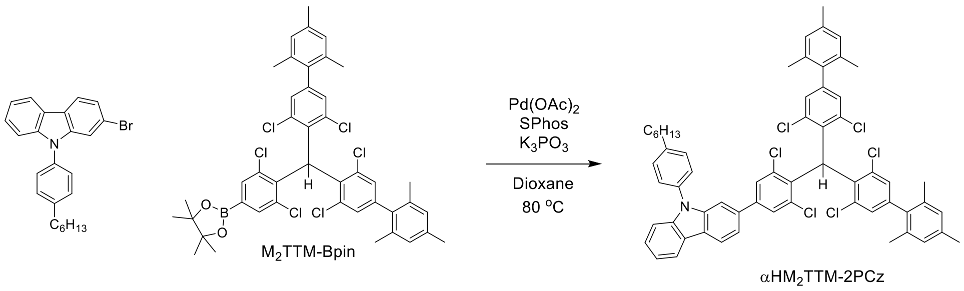

2-(4-(Bis(3,5-dichloro-2',4',6'-trimethyl-[1,1'-biphenyl]-4-yl)methyl)-3,5-dichlorophenyl)-9-(4-hexylphenyl)-9*H*-carbazole (αHM$_2$TTM-2PCz). 2-Bromo-9-(4-hexylphenyl)-9*H*-carbazole (0.050 g, 0.12 mmol, 1 equiv.), M$_2$TTM-Bpin (0.120 g, 0.15 mmol,

1.2 equiv.), $Pd(OAc)_2$ (0.0008 g, 0.0037 mmol, 0.03 equiv.), SPhos (0.0038 g, 0.0092 mmol, 0.075 equiv.) and K_3PO_4 (0.038 g, 0.18 mmol, 1.4 equiv.) were added into a microwave vial and the vial was subjected to three vacuum/Ar gas refill cycles. Anhydrous 1,4-dioxane (1 mL) was added and the mixture was heated at 80 °C oil bath for 24 h. After cooling to RT, the mixture was diluted with hexane, extracted with water (3 × 200 mL) and the organic phase was dried over anhydrous $MgSO_4$. Solvent was removed under vacuum and the crude product was purified with column chromatography over silica gel, by gradually increasing the eluent polarity from hexane to 10% (v/v) DCM in hexane. Solvent was removed and the solids were sonicated in MeOH (3 × 5 min) and washed with hexane/MeOH 1:1 (v/v) mixture. The solids were collected each time by centrifuging and the solvent was removed by pipetting. Finally, the solids were dried in vacuum and the target compound was collected as white solid (0.090 g, 72%). ^{1}H NMR (400 MHz, $CDCl_3$) δ 8.18 (dd, J = 15.3, 7.8 Hz, 2H), 7.67 (d, J = 2.0 Hz, 1H), 7.59–7.36 (m, 9H), 7.30 (ddd, J = 8.1, 6.8, 1.4 Hz, 1H), 7.18 (dd, J = 4.9, 1.8 Hz, 2H), 7.04 (dd, J = 3.7, 1.8 Hz, 2H), 7.00 (s, 1H), 6.94 (s, 4H), 2.81–2.72 (m, 2H), 2.33 (s, 6H), 2.07 (d, J = 4.3 Hz, 12H), 1.81–1.71 (m, 2H), 1.51–1.34 (m, 6H), 0.93 (t, J = 7.2 Hz, 3H). ^{13}C NMR (100 MHz, $CDCl_3$) δ 142.80, 142.46, 142.00, 141.88, 141.56, 137.69, 137.58, 137.51, 137.38, 137.05, 136.83, 136.79, 136.05, 135.87, 135.72, 135.71, 135.66, 134.78, 134.38, 134.17, 134.07, 131.06, 130.99, 130.02, 129.29, 129.24, 128.88, 128.16, 127.09, 126.25, 123.33, 122.73, 120.75, 120.41, 120.05, 119.03, 109.99, 107.99, 50.53, 35.77, 31.74, 31.37, 29.15, 22.63, 21.04, 20.59, 20.57, 20.54, 14.12. TOF-MS-ASAP^{+} Calcd. for $[C_{61}H_{54}Cl_6N]^{+}$: 1010.2387. Found: *m/z* = 1010.2403. EA Calcd. for $C_{61}H_{53}Cl_6N$: C, 72.34; H, 5.27; Cl, 21.00; N, 1.38. Found: C, 72.32; H, 5.23; N, 1.37.

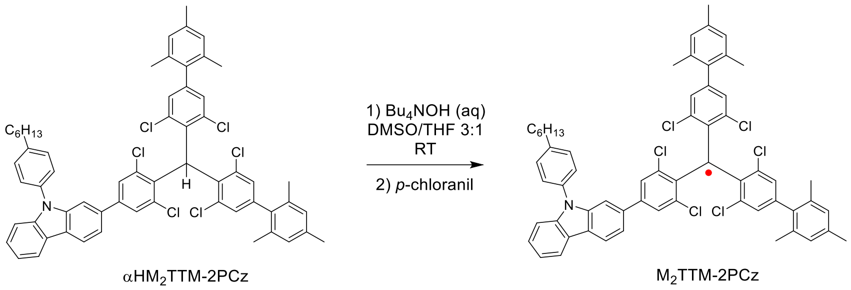

2-(4-(Bis(3,5-dichloro-2',4',6'-trimethyl-[1,1'-biphenyl]-4-yl)methyl)-3,5-dichlorophenyl)-9-(4-hexylphenyl)-9*H*-carbazolyl radical (M_2TTM-2PCz). αHM_2TTM-2PCz (0.0400 g, 0.039 mmol, 1 equiv.) was added into a round-bottom flask and the flask was subjected to three vacuum/Ar gas refill cycles. Anhydrous THF (2 mL) was added to dissolve all starting material followed by addition of anhydrous DMSO (6 mL). The mixture was bubbled with Ar gas for 15 min and covered carefully from light. In the darkness, 40% Bu_4NOH (aq) (0.05 mL, 0.079 mmol, 2 equiv.) (bubbled with Ar gas for 15 min prior to use) was added and the mixture was stirred for 6 h. Following the addition of base, the initially colorless reaction mixture quickly acquired dark blue color. *p*-Chloranil (0.0243 g, 0.099 mmol, 2.5 equiv.) was added and the mixture was stirred another 6 h, quickly changing color to dark yellow/green. Under minimal amount of ambient light, the mixture was diluted with hexane, extracted with water (3 × 200 mL) and the organic phase was dried over anhydrous $MgSO_4$. Solvent was removed under vacuum and the crude product was purified with column chromatography over silica gel, by gradually increasing the eluent polarity from hexane to 5% (v/v) DCM in hexane. Solvent was removed and the solids were sonicated in MeOH for 5 min. The solids were collected by centrifuging and MeOH was removed by pipetting. Finally, the solids were dried in vacuum and the target compound was collected as dark green solid (0.033 g, 83%). The radical was stable under ambient air in both solution and solid state, but when stored for longer times the materials were kept under inert gas. TOF-MS-ASAP$^+$ Calcd. for $[C_{61}H_{53}Cl_6N]^+$: 1009.2309. Found: m/z = 1009.2289. EA Calcd. for $C_{61}H_{52}Cl_6N^•$: C, 72.41; H, 5.18; Cl, 21.02; N, 1.38. Found: C, 72.31; H, 5.19; N, 1.40.

NMR monitoring of radical conversion and reverse α-hydrogenation

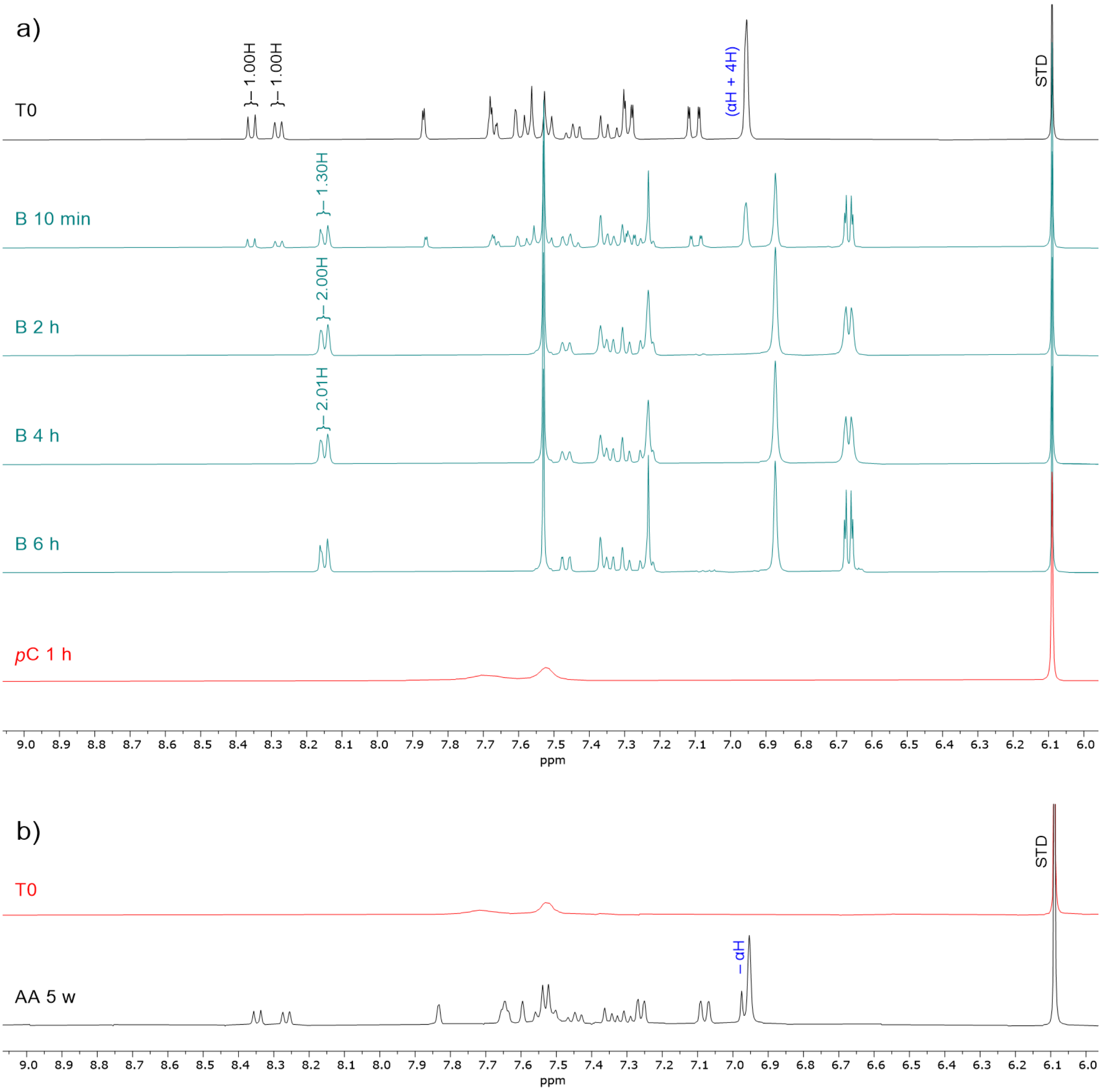

Supplementary Figure 25: Radical conversion and α-hydrogenation of M_2TTM-2PCz radical, as monitored by NMR. **a**, Deprotonation of the αH precursor (40% Bu_4NOH, aq, 2 equiv.) followed by one-electron oxidation (*p*-chloranil, 2.5 equiv.) in DMSO-d_6/THF-d_8 3:1 (v/v) mixture. ^{1}H NMR spectra of the αH precursor at time-zero (T0) before deprotonation (black line), 10 min, 2 h, 4 h and 6 h after addition of base (B) showing formation of the carbanion (teal lines) and 1 h after addition of *p*-chloranil (*p*C) showing formation of the radical (red line), as indicated in the legend. Selected peak integrals are shown to illustrate complete deprotonation and formation of anionic species within 4 hours. **b**, α-Hydrogenation of the radical (L-ascorbic acid, aq, 20 equiv.) in DMSO-d_6/THF-d_8 1:1 (v/v) mixture. ^{1}H NMR spectra of the radical before α-hydrogenation (T0, red line) and 5 weeks after addition of L-ascorbic acid (AA) showing slow formation of the α-hydrogenated product (black line), as indicated in the legend. No carbanion species was observed in the reverse reaction. The αH signal is overlapping with another 4H signal in (**a**) and it is only resolved in DMSO-d_6/THF-d_8 1:1 (v/v) mixture in (**b**) and in $CDCl_3$. All spectra have been referenced against 1,3,5-trimethoxybenzene (^{1}H, 6.09 ppm) as the internal standard.

NMR spectra

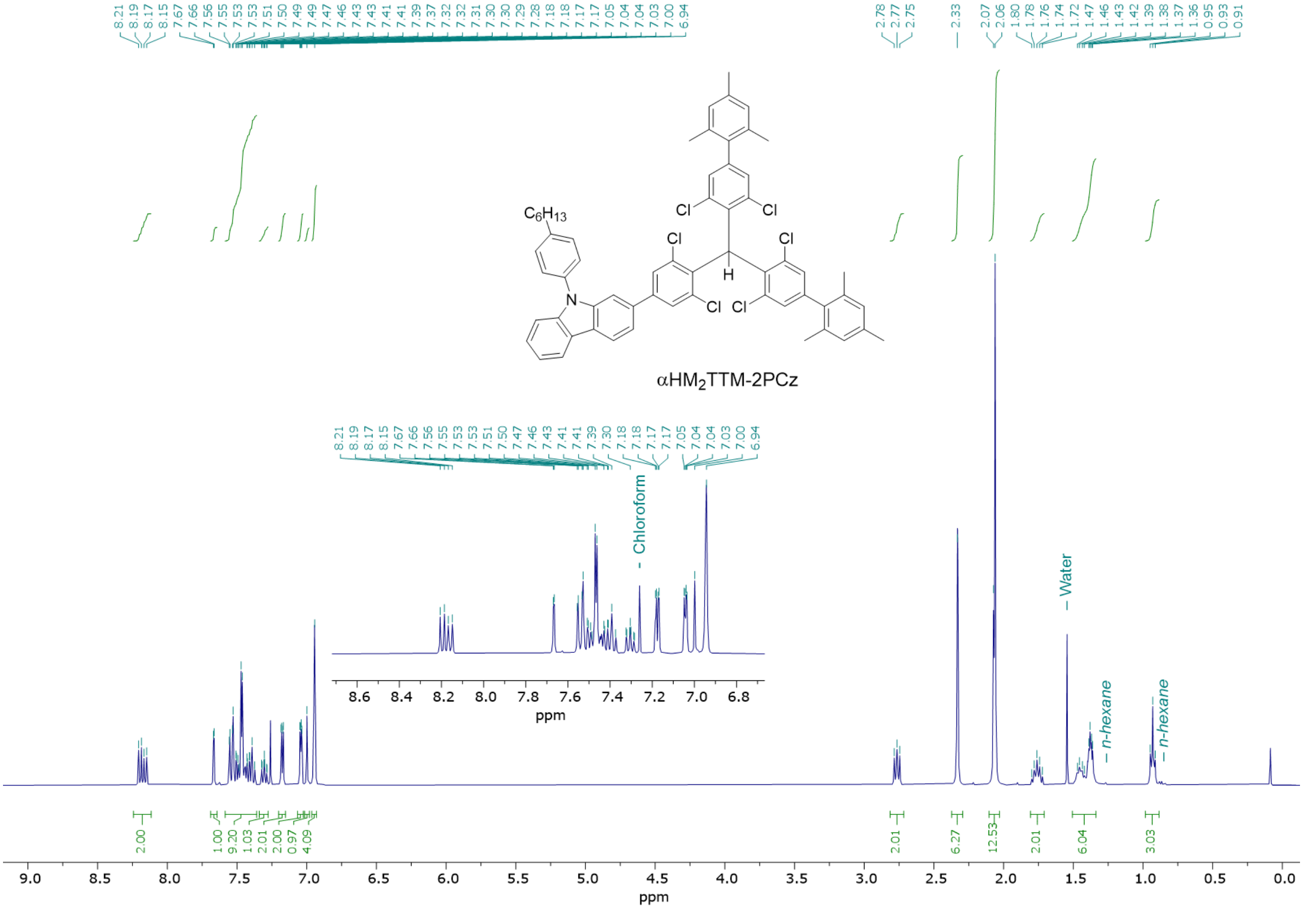

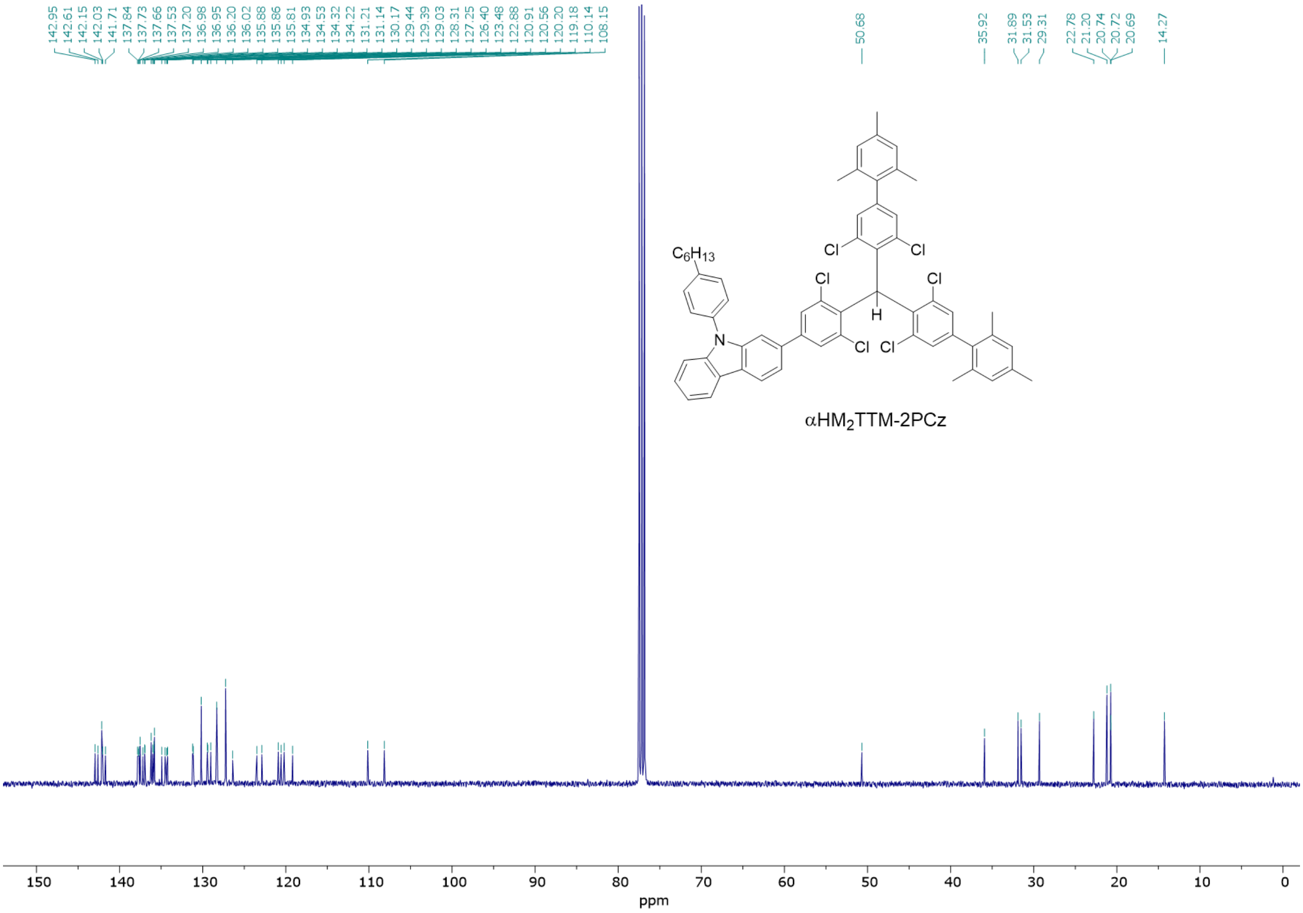
142.95
142.61
142.15
142.03
141.71
137.84
137.73
137.66
137.53
137.20
136.98
136.95
136.20
136.02
135.88
135.86
135.81
134.93
134.53
134.32
134.22
131.21
131.14
130.17
129.44
129.39
129.03
128.31
127.25
126.40
123.48
122.88
120.91
120.56
120.20
119.18
110.14
108.15
50.68
35.92
31.89
31.53
29.31
22.78
21.20
20.74
20.72
20.69
14.27
C_6H_{13}
N
Cl
H
αHM2TTM-2PCz
150
140
130
120
110
100
90
80
70
60
50
40
30
20
10
0
ppm

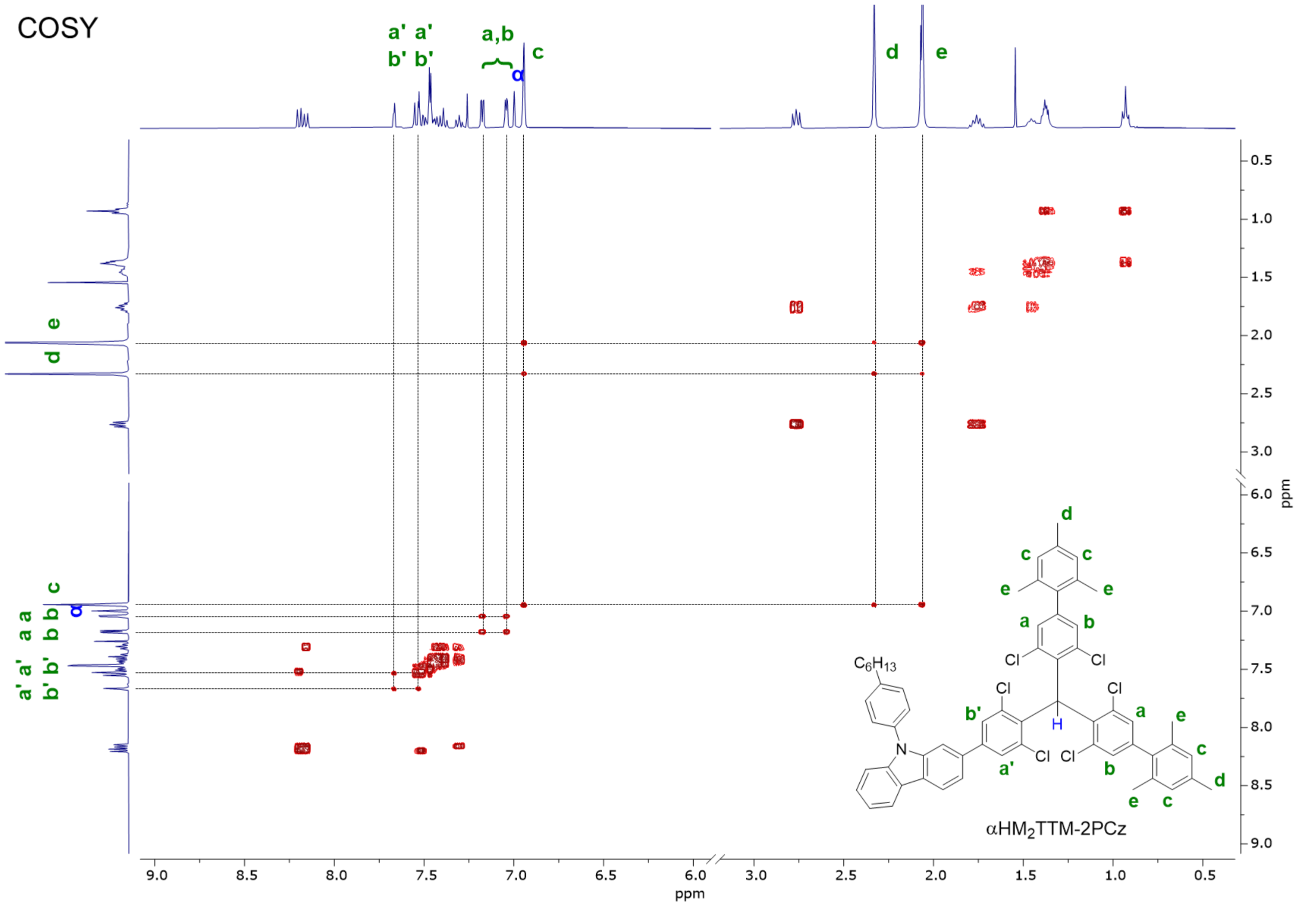
COSY
a' a'
b' b'
a,b
α
c
d
e
C_6H_{13}
N
Cl
H
αHM2TTM-2PCz
9.0
8.5
8.0
7.5
7.0
6.5
6.0
3.0
2.5
2.0
1.5
1.0
0.5
ppm

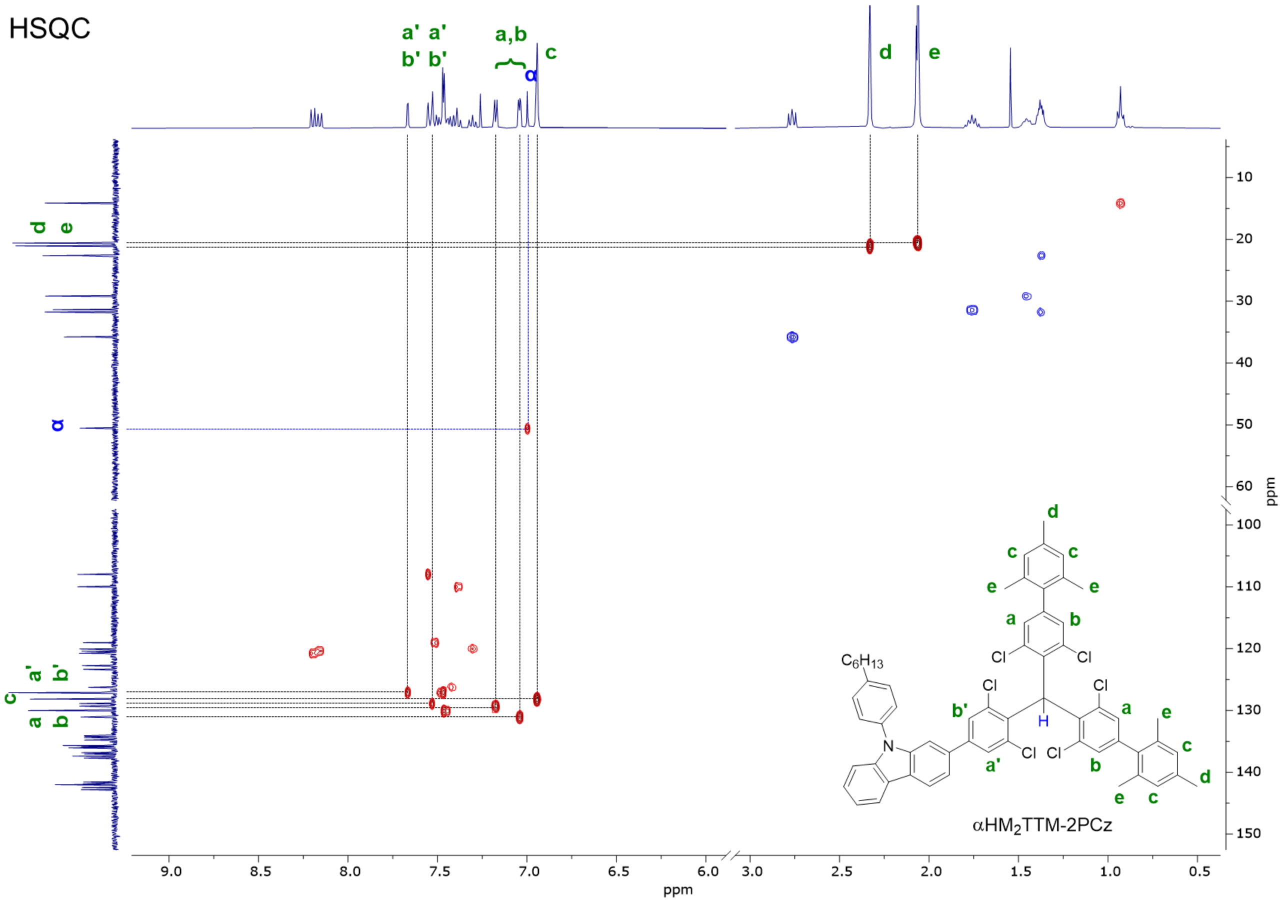
HSQC
a' a'
b' b'
a,b
c
α
d
e
C6H13
N
Cl
H
αHM2TTM-2PCz
ppm

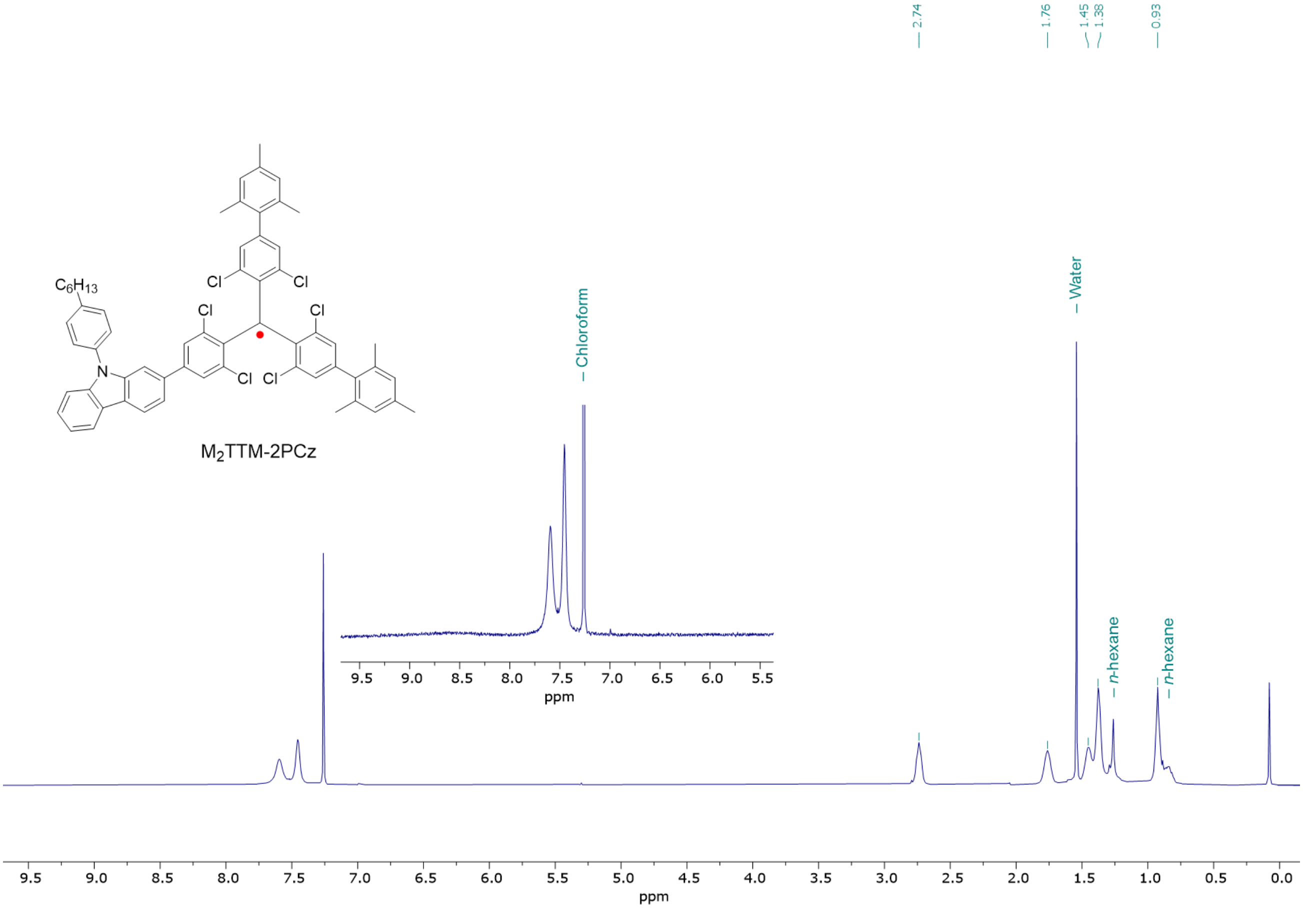
2.74
1.76
1.45
1.38
0.93
C6H13
N
Cl
M2TTM-2PCz
Chloroform
Water
n-hexane
n-hexane
ppm

Section 18: **mode-resolved Non-radiative loss probed by vibrational coherence**

In the current section we have discussed how the vibrational modes with near zero reorganization energy which behaves as spectator modes in the nuclear coordinate of non-radiative transition, cannot be efficiently impulsively generated in the photo-excited wavepacket.

18.1. Non-radiative loss: a mode-resolved picture

Non-radiative transitions from the electronic excited (S_1) to the ground (S_0) state promoted by the vibrational normal modes can be described semi-classically by the energy-gap law[28,29]. We have applied this approach which is also similar to that used to understand the non-radiative recombination in the organic photovoltaic systems by K. Vandewal[30,31] and coworkers and J. Nelson and coworkers[32] where they adopted a model introduced by Jortner[33] considering the solvents/surrounding modes can be thermally activated while the molecular modes are frozen. Using this concept, the non-radiative rate can be described under the approximation of Fermi's golden rule and Born-Oppenheimer approximation.

$$k_{nr} \propto V^2 FC$$

V is the electronic coupling between the ground and excited electronic state and the FC is the Frank-Condon wavefunction overlap weighted density of states.

$$FC = (4\pi\lambda_S k_B T)^{-0.5} \sum_{n=0}^{\infty} e^{-S} S^n (n!)^{-1} exp^{\left\{-\frac{(g - nh\bar{\nu}_v - \lambda_S)^2}{4\lambda_S k_B T}\right\}}$$

Where $e^{-S} S^n (n!)^{-1}$ describes the wavefunction overlap between 0th vibration state of the excited electronic state and the nth vibrational state of the ground electronic state. S is the Huang-Rhys factor.

The mode-resolved rate equation for the non-radiative transitions derived by Jortner and Englman in the weak electronic coupling limit where no curve crossing happens between the electronic surfaces can be represented as:

$$k_{nr} = \frac{C^2 (2\pi)^{\frac{1}{2}}}{\hbar(\hbar\omega_i \Delta E)^{\frac{1}{2}}} \exp^{-\left[\frac{\Delta E}{\hbar\omega_i}\left\{\ln\left(\frac{\Delta E}{\sum_i \lambda_i}\right) - 1\right\}\right]}$$

Where ω_i is frequency of the 'i'-th mode with reorganization energy λ_i. Chou and coworkers[34,35] have also used this formalism to understand and reduce non-radiative

loss in metal-organic emitters. This equation is frequently referred to the Energy Gap Law.

Here, we categorize all the high and low frequency normal modes into two distinct categories:

1. Spectator modes for non-radiative decay process: These modes exhibit a reorganization energy (λ_i) that is approximately equal to zero. Additionally, the normal coordinates of these modes are orthogonal to the nuclear coordinates of the nonradiative transition. Displacement along these modes has minimal impact on the Frank-Condon overlap, which represents the wavefunction overlap between the vibrational states of the ground and excited states. Consequently, these modes do not significantly contribute to the non-radiative transition.
2. Driving modes for non-radiative decay process: In contrast, driving modes are characterized by non-zero reorganization energies (λ_i) for the nonradiative transition. Along the coordinates of these modes, there is a strong Frank-Condon overlap, signifying their significant involvement in the non-radiative transition process.

In Supplementary Fig. 26-a, we present an analytical model for the evolution of the FC overlap along a vibrational coordinate with the frequency ω, to understand the non-radiative loss behaviour. This analysis follows ref[31] where Benduhn *et al*. performed such an analytical model for the parameter ΔE. We are assuming three different scenarios:

1. non-displaced with low energy-gap (red)
2. non-displaced with high-energy-gap (blue)
3. displaced with high-energy-gap (green).

This visualization illustrates an analytical comprehension of the relationship between non-radiative losses and the Frank-Condon overlap concerning two key parameters: 1. energy-gap (ΔE), 2. dimensionless displacement along a mode (Δ).

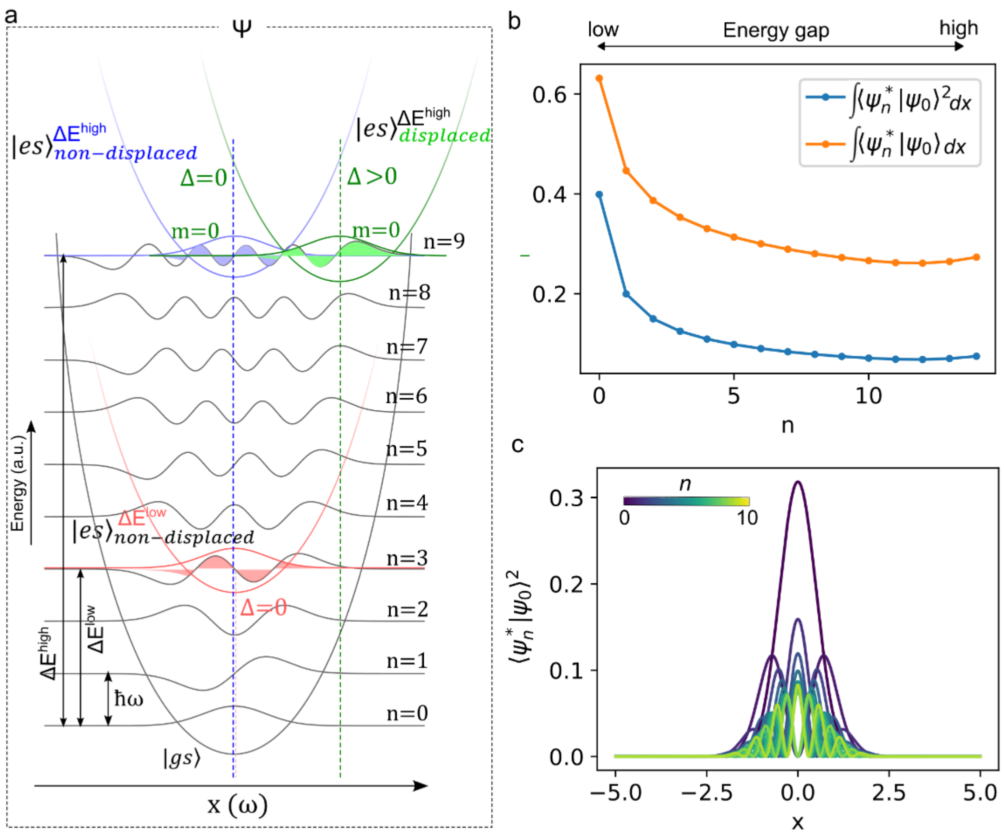

Supplementary Fig. 26: **a,** Potential energies of the ground state, $|gs\rangle$ and excited state, $|es\rangle$ as a function of the dimensionless displacement (Δ) along the reaction coordinate a vibrational mode with frequency ω. The $|es\rangle$ surface drawn in red has zero displacement and blue corresponds to the displaced potential surface with respect to the $|gs\rangle$. "m" represents the vibrational states in the $|es\rangle$ manifold and "n" represents the vibrational states in the $|gs\rangle$ manifold. The vibrational wavefunctions (ψ) plotted with the Hermite polynomials. **b,** $\int_{-x}^{x}\langle\psi_n^*|\psi_0\rangle^2 dx$ (blue curve) and $\int_{-x}^{x}\langle\psi_n^*|\psi_0\rangle dx$ (orange curve) plotted against the vibrational quantum number (n) of the ground state for the non-radiative transition from the i=0 ($|\boldsymbol{es}\rangle$) as representative of Frank-Condon factor.; **c,** $\langle\psi_n^*|\psi_0\rangle^2$ plotted against nuclear coordinate for different vibrational quantum number (n).

The vibrational states in the excited state manifold are denoted by 'm' ($m = 0$, in this case) and the vibrational states in the ground electronic state manifold are denoted by 'n'. As visualised in the Supplementary Fig. 26 b,c, the FC overlap (in the form of $\int_{-x}^{x}\langle\psi_n^*|\psi_{m=0}\rangle^2 dx$, $\int_{-x}^{x}\langle\psi_n^*|\psi_{m=0}\rangle dx$, $\langle\psi_n^*|\psi_{m=0}\rangle^2$) increases with lowering the value of the vibrational quantum number, n. This is consistent with the energy-gap law, which predicts the enhancement in the non-radiative loss with lowering the energy-gap, and also with the analytical model of Ref[31].

The effect of the dimensionless displacement (Δ) along any mode is analytically studied with the model under the harmonic approximation. As illustrated in the Supplementary Fig. 27 a-d, we present the wavefunction overlap in the form of $\langle\psi_n^*|\psi_{m=0}\rangle$ and $\langle\psi_n^*|\psi_{m=0}\rangle^2$ for n=5 (symmetric state) and n=8 (asymmetric state) and we can see a systematic increase in the FC wavefunction overlap with increasing the Δ. $\int_{-x}^{x}\langle\psi_n^*|\psi_0\rangle dx$ calculated for n=2,3,4,10 (Supplementary Fig. 27 e-h) predicts higher non-radiative rates with increasing the displacement.

Collectively, our analytical model, operating under the harmonic approximation, demonstrates that reducing the displacement between the minima of electronic surfaces, results in a decrease in vibrational Frank-Condon overlap, consequently supressing non-radiative decay.

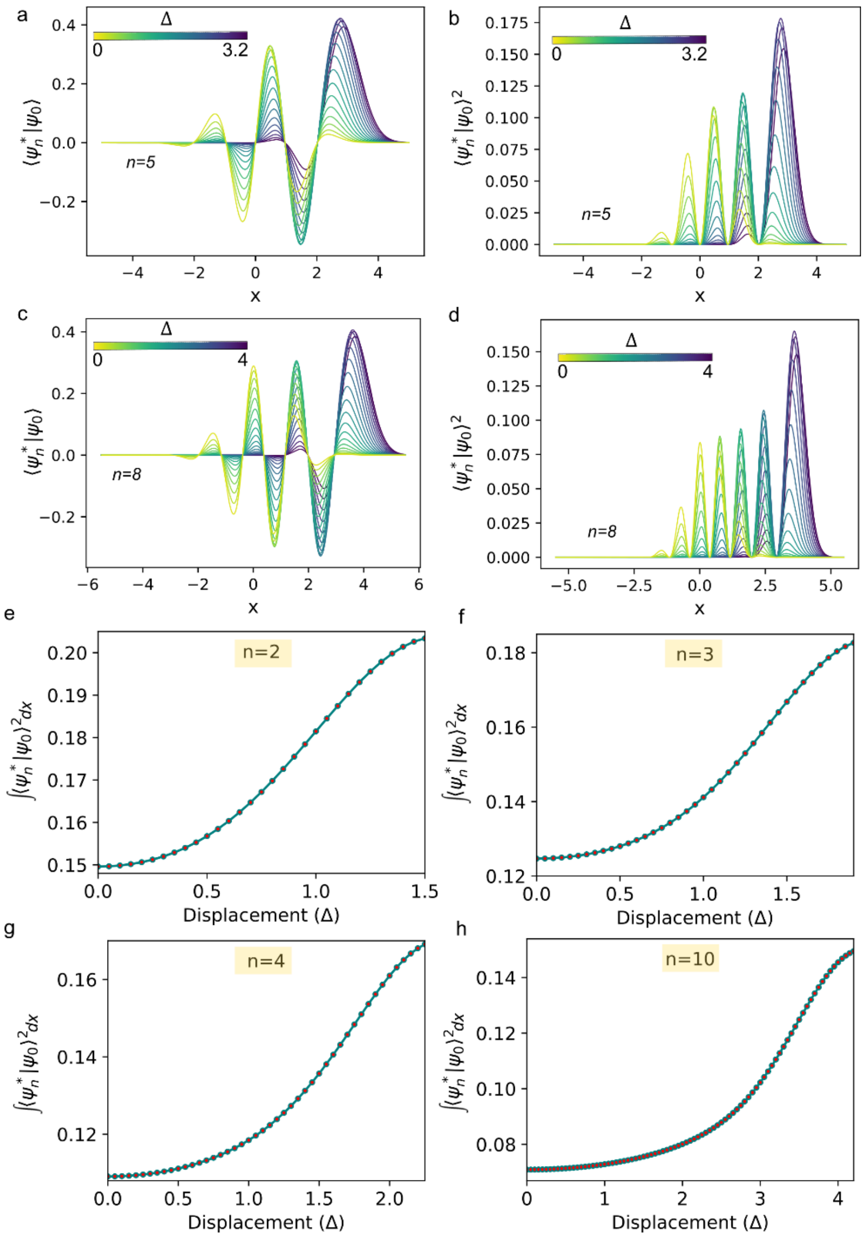

Supplementary Fig. 27: **a,b,** $\langle\psi_n^*|\psi_0\rangle$ and $\langle\psi_n^*|\psi_0\rangle^2$ for the nonradiative transition between i=0 ($|\boldsymbol{es}\rangle$) to **n** =5 ($|\boldsymbol{gs}\rangle$, **asymmetric state**) in a and b respectively. **c,d,** $\langle\psi_n^*|\psi_0\rangle$ and $\langle\psi_n^*|\psi_0\rangle^2$ for the nonradiative transition between i=0 ($|\boldsymbol{es}\rangle$) to **v** =8 ($|\boldsymbol{gs}\rangle$, **symmetric state**) in c and d respectively.; **e,f,g,h,** $\int_{-x}^{x}\langle\psi_n^*|\psi_0\rangle dx$ calculated against unitless displacement (Δ) along the mode in the harmonic limit for the non-radiative transition from i=0 ($|\boldsymbol{es}\rangle$) to e, **n** =2 ($|\boldsymbol{gs}\rangle$; **f, n** =3 ($|\boldsymbol{gs}\rangle$; **g n** =4 ($|\boldsymbol{gs}\rangle$; **h, n** =10 ($|\boldsymbol{gs}\rangle$;

18.2. Vibrational coherence generated on the excited state manifold with the impulsive vibrational spectroscopy

In the electronic excited state (S_1) manifold, the vibrational ground state is represented as $\psi_{i=0}(x)$ and the first excited vibrational states are $\psi_{i=1}(x)$. After impulsive photoexcitation by

a broadband laser pulse from the electronic ground state, multiple vibrational states will be populated, leading to, a newly generated non-stationary states which can be represented as:

$$\psi(r,t) = c_{i=0}(t)e^{-i\omega_0 t}\psi_{i=0}(r) + c_{i=1}(t)e^{-i\omega_1 t}\psi_{i=1}(r) + \cdots$$

Where $c_{i=0}$, $c_{i=1}$, corresponds to the contribution of the each vibrational states to the non-eigen state. Hence, for a narrow-band, non-impulsive excitation (no coherent superposition of states) then $c_{i=0} = 1$, $c_{i=1} = 0$. It is noteworthy that the $\psi(r,t)$ has an exponential damping term as well which stands for the dephasing.

The time-dependent molecular polarization can be described as -

$$P(t) = \langle \psi(r,t)|\mu|\psi(r,t)\rangle$$

Where μ is the dipole moment operator.

$$P(t) = \left\langle c_{i=0}(t)e^{-i\omega_0 t}\psi_{i=0}(r) + c_{i=1}(t)e^{-i\omega_1 t}\psi_{i=1}(r) \middle| \mu \middle| c_{i=0}(t)e^{-i\omega_0 t}\psi_{i=0}(r) + c_{i=1}(t)e^{-i\omega_1 t}\psi_{i=1}(r)\right\rangle$$

$$P(t) = c_{i=0}^{*}c_{i=1}\mu_{01}e^{-i(\omega_1-\omega_0)t} + c_{i=1}^{*}c_{i=0}\mu_{10}e^{-i(\omega_0-\omega_1)t}$$

$$P(t) = \mu_{01}(c_0^{*}c_1e^{-i(\omega_1-\omega_0)t} + c_1^{*}c_0e^{i(\omega_1-\omega_0)t})$$

The macroscopic polarization can be represented as $P_N(t) = N.P(t)$, where N is the number of molecules[36]. Hence, the vibrational coherence generated by the superposition of two vibrational states can oscillate with $(\omega_1 - \omega_0) = \omega^{|es\rangle}$. The oscillatory time dependent change in the macroscopic polarization will vanish if the energetic bandwidth of the excitation laser source is lower than $\omega^{|es\rangle}$. Another key factor for impulsive generation of the wavepacket is during the direct impulsive excitation, the excited surface must have displacement (Δ) with respect to the ground state[37–39].

As a result nuclear wavepacket motion in the multidimensional vibration co-ordinate can be used as a probe for exciton-vibrational coupling. Similar displaced harmonic oscillator model for the band edge exciton transition is also envisioned to quantify the exciton-vibration coupling in inorganic semiconductors by S. Ruhman and coworkers[40], Tze Chien Sum and coworkers[41]. In such impulsive excitation, electron−phonon coupling strengths are represented by a set of parameters [Δ, S, λ]. Δ is a dimensionless displacement of the normal coordinate (as discussed above), S is the Huang−Rhys parameter, and λ is the reorganization energy.

Both S and λ exclusively dependent on the displacement (Δ) as follows:

$$S = \frac{\Delta^2}{2}; \lambda = \hbar\omega S$$

where ω is the frequency of the optical phonon. λ ($\hbar\Delta\omega$) can be calculated from impulsive vibrational spectroscopy data using $A_{OSC} = \left(\frac{d\ OD}{d\omega}\right)\Delta\omega$, where OD is the optical density of the sample and A_{OSC} is the amplitude of the oscillations. A_{OSC} of any vibrational modes can be obtained by fitting the residuals to a damped sine function. Relative A_{OSC} between different modes for comparison purposes, can be obtained from the relative FFT amplitudes.

Taken together , the spectator modes for a non-radiative transition in the weak coupling limit, which has near zero λ (Δ) cannot efficiently be impulsively generated and are therefore not observed in our experiments. Whereas the driving modes which have larger λ (Δ), Δ and S, can be efficiently generated in the impulsive wavepacket.

Section 19: **Impulsive vibration spectroscopy of TTM-TPA in variable solvent polarity**

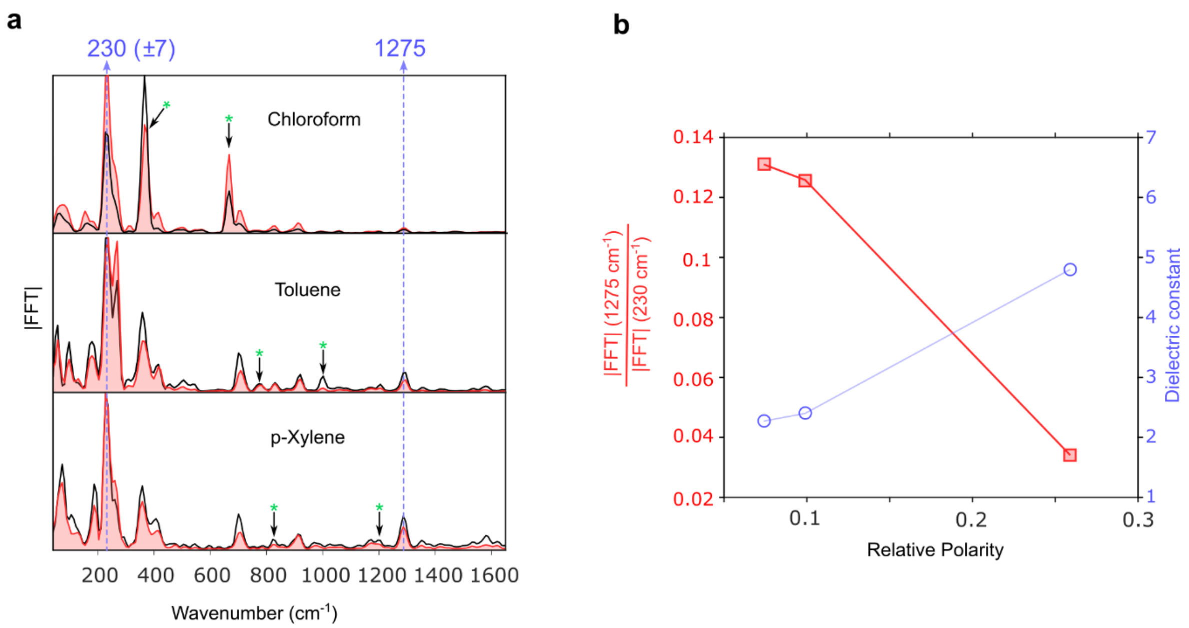

Supplementary Fig. 28: Solvent dependent IVS (P1) on TTM-TPA. **a,** IVS data of the TTM-TPA in different solvents (solvent polarizability: p-Xylene<Toluene<Chloroform). Integrated Fourier transformed spectra for chloroform, toluene, p-Xylene (Integrated over $\lambda = 530 - 750\ nm$ (red), $\lambda = 650 - 850\ nm$ (black)) **b,** Intensity ratio of $|FFT|^2$(1275 cm^{-1}) to $|FFT|^2$(230 cm^{-1}) plotted against the relative polarity of the solvent used (red data). For reference dielectric constant of the solvent is also plotted (blue). Modes that are asterisked are off-resonant contribution of the solvent modes.

To further investigate how the degree of charge-transfer plays a role in the radical emitter system, we performed resonant broadband impulsive vibrational spectroscopy on TTM-TPA in 3 solvents with varying polarity (relative polarity order: p-Xylene<Toluene<Chloroform), using P1 to impulsively excite the $D_0 \rightarrow D_1$ transition.

As shown in Supplementary Fig. 28a, we find that high frequency modes (e.g. 1275 cm^{-1}) for the most polar solvent (Chloroform) exhibit minimum intensity relative to the low frequency modes. In the least polar solvent (p-Xylene), the same high-frequency mode shows a higher

intensity relative to the low-frequency modes. The trend showcased in Supplementary Figure 28 b aligns with a systematic decrease in the ratio of the contribution of the representative high-frequency mode at 1275 cm^{-1} to the contribution of the representative low-frequency mode at 230 cm^{-1} as the solvent's relative polarity and dielectric constant increase. These findings further support that the suppression of exciton coupling to high-frequency, localized C-C stretching modes occurs when the exciton exhibits charge-transfer characteristics involving spatially-separated electron-hole pairs.

Within the framework of the 'energy-gap law' rate expression, it is crucial to recognize that the non-radiative rate (Knr) is influenced by both, the emission energy gap (ΔE) and the frequency of strongly coupled vibrational modes.

As illustrated in Supplementary Fig. 29 a, the impact of the energy-gap law on Knr is significantly reduced in radical systems compared to e.g. NFA systems, due to the decoupling of high-frequency modes. However, the energy gap law still applies! Consequently, Knr depends on two contrasting effects: a) a decreasing trend in Knr due to the reduced coupling of high-frequency modes with increasing charge-transfer character and b) an increasing trend in Knr resulting from the lower energy gap due to a higher Stokes shift in more polar solvents.

This complication is further supported by previous work[42] on a similar class of TTM-based radicals, where the donor was pyridoindolyl (PyID) instead of triphenylamine (TPA), where it was found that Knr is largely independent of solvent polarity, as depicted in Supplementary Fig. 29 b. Therefore, the interplay of these two competing effects on non-radiative losses with varying solvent polarity can elucidate the observed trends.

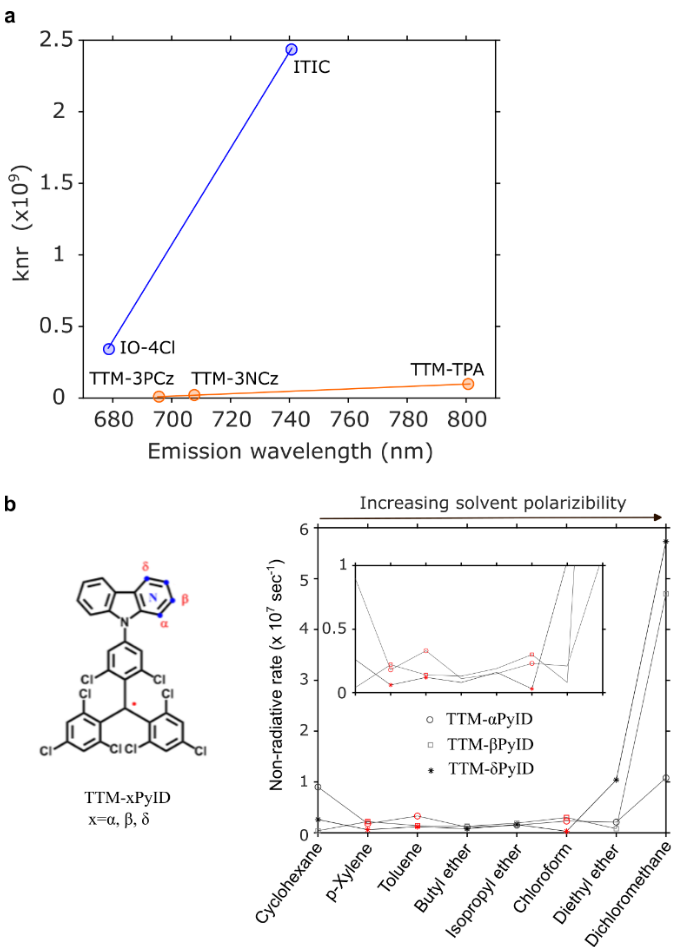

Supplementary Fig. 29: a, Non-radiative rate for NFA-systems with similar structure (IO-4Cl, o-IDTBR) and radical systems (TTM-3PCz, TTM-3NCz and TTM-TPA). b, Non-radiative rate plotted for TTM-Carbazole radicals TTM–pyridoindolyl derivatives: TTM–αPyID, TTM–βPyID and TTM–δPyID in different solvents with variable polarity. (the data points that are highlighted in red are the solvents which are used for solvent-dependent IVS of TTM-TPA). This dataset are taken from the reference[42].

Another technical aspects of this experiments that is noteworthy are as follows:

1. Despite retaining the same P1 pump spectra for all experiments, variations arise in the absorption cross-section between absorption spectra and pump spectra due to the solvatochromic shift (Supplementary Fig. 30). Hence, the coefficient $c_{i=1}(t)$ in the wavepacket equation, will exhibit sample-to-sample variations in the impulsive population of a high-frequency mode leading to highest impulsive population of the high-frequency mode for the solvent with broadest absorption cross-section.

   $\psi(r,t) = c_{i=0}(t)e^{-i\omega_0 t}\psi_{i=0}(r) + c_{i=1}(t)e^{-i\omega_1 t}\psi_{i=1}(r)$ (wavepacket equation).

   Intriguingly, in Chloroform the overlap between pump and absorption spectra is broadest. Therefore, if we could correct for this effect, the trend depicted in Supplementary Figure 28 would gain even more significance.

2. The differences in the absorption cross-sections in different solvents, leads to minute variation in the pump beam's penetration depth from the front surface of the cuvette. This variation leads to varying pulse dispersion, with the most significant dispersion

occurring in less absorbing samples. Consequently, it's crucial to recognize that the effective time-resolution will slightly differ across various samples, particularly in the context of quantifying the absolute intensity of high-frequency modes. To minimise this effect we performed our solvent dependent IVS experiments in ultrathin (200 micrometre path length cuvette).

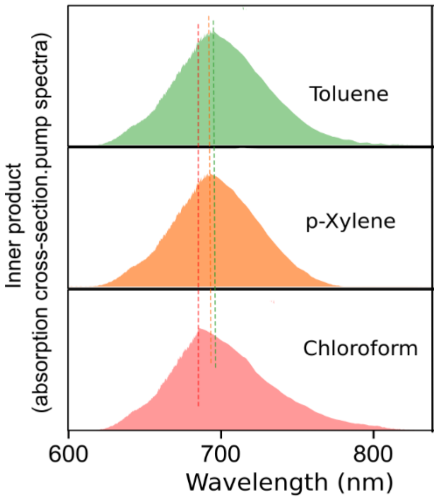

Supplementary Fig. 30: The plots depicts the overlapped area between the P1-pump spectra and the absorption spectra of TTM-TPA in different solvents

Section 20: **Extended discussion on dependence of exciton-vibration coupling on degree of charge transfer character**

Following ref[43], the energy of a local exciton may be written as:

$$E_{LE} = \Delta\varepsilon - W + I + J,$$

where in a simple picture one can consider $\Delta\varepsilon$ to be equal to the HOMO-LUMO gap of a closed-shell molecule (hence directly related to the IP and EA through Koopman's theorem), I, J so-called excitation transfer integrals, and W the *on-site* screened Coulomb interaction.

On the other hand, a CT exciton has an energy which may be written as:

$$E_{CT} = \Delta\varepsilon - \overline{W},$$

where $\overline{W}$ the inter-molecular screened Coulomb interaction, which in this case represents the Coulomb interaction between neighbouring donor-acceptor moieties.

Let us now consider a finite displacement δu along a given vibrational mode of frequency ω. The coupling of the excited states to the vibration is proportional to the first derivative of their

energy along δu, therefore, if g is the coupling constant of each state to the mode (LE: Local exciton (non-charge transfer), CT: Charge transfer exciton):

$$g_{LE} - g_{CT} \cong \frac{\delta \bar{W}}{\delta u} - \frac{\delta W}{\delta u},$$

Where we have assumed that the change of the transfer integrals is small.

There are two cases to distinguish:

1. Low-frequency vibrational mode
   In this case the displacement δu results in changes in the distance between the D-A moieties, and does not significantly alter the intramolecular structure. Therefore, $\frac{\delta W}{\delta u} \cong 0$ and $\frac{\delta \bar{W}}{\delta u}$ remains finite, hence $|g_{LE}| < |g_{CT}|$.
2. High-frequency vibrational mode
   In this case the displacement δu results in changes in the internal structure of the D-A moieties, while their intermolecular distance may be considered to remain the same. Therefore, here we have $\frac{\delta \bar{W}}{\delta u} \cong 0$ and $\frac{\delta W}{\delta u}$ remains finite, resulting in $|g_{LE}| > |g_{CT}|$.

Therefore, overall, this simple picture suggests that a local exciton is expected to couple preferentially to high-frequency vibrations, while excitons with strong charge transfer character will exhibit stronger coupling to low-frequency vibrations. This is consistent with fully first-principles results in organic molecular crystals[44,45].

Section 21: **Extended discussion on vibrational coupling and non-radiative decay in non-fullerene acceptors**

As presented in the Extended Data Fig.2, all the NFA molecules (Supplementary Figure 34 for chemical structures) that we studied show much stronger coupling to the high-frequency (>1000 cm^{-1}) in comparison to the radical and TADF molecules studied. This observation can be explained as the absence of both - a) pure non-bonding character in the hole and electron accepting levels, b) disjoint electron and hole wavefunction promoted by charge-transfer character and strong structural twisting.

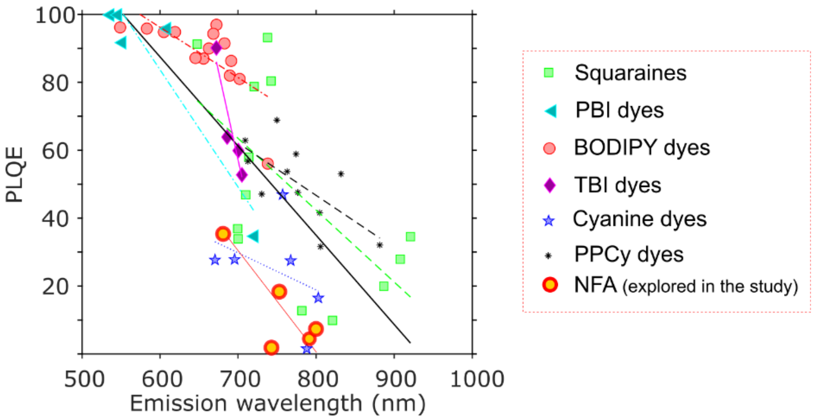

Supplementary Fig. 31 : a, PLQE trends by the series organic chromophores and NFAs studied here plotted against emission wavelength. Data of the other chromophores are taken from ref[46,47]. The solid black line represents the combined dataset.

We note that in NFAs, the $S_0 \rightarrow S_1$ transition exhibits weak charge-transfer character, primarily induced by push-pull effect by the heteroatoms[48]. This push-pull type effect predominantly contribute to the reduced bandgaps in NFAs. However, this charge-transfer character in the $S_0 \rightarrow S_1$ transition of NFAs is notably weaker than for highly twisted, non-planar TADFs and the radical's lowest energy electronic transition. Consequently, radicals (such as TTM-3PCz, TTM-3NCz, TTM-TPA, M_2TTM-3PCz) and TADFs (APDC-DTPA, 4CzIPN) studied exhibit lower absorption coefficients with spatially-separated electron-hole pairs, in contrast to NFAs, which display a more substantial overlap between electron and hole wavefunctions (as illustrated in Supplementary Figure 14) and are chemically engineered to efficiently absorb photons for use in organic photovoltaic (OPV) applications. As a result of the stronger coupling to the high-frequency modes exceeding 1000 cm^{-1} (similar to Rhodamine-6G and rr-P3HT, main), PLQE trends in NFA molecules closely adhere to the energy-gap law, similar to conventional dyes (refer to Supplementary Figure 31 for details).

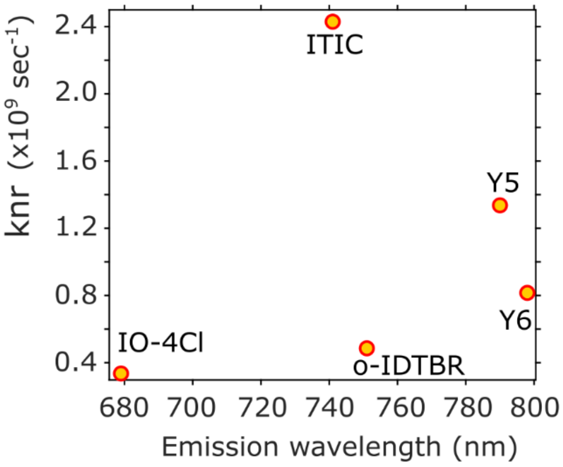

Supplementary Fig. 32: Non-radiative rates plotted for the NFAs studied.

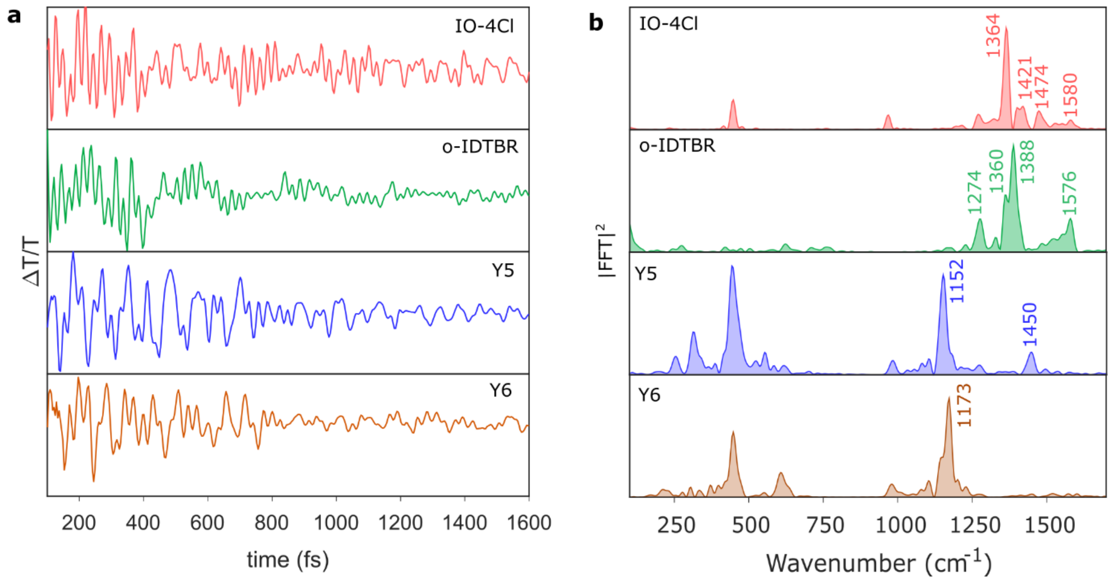

Supplementary Fig. 33: a, vibrational coherence extracted from the IVS experiment (integrated in the photo-induced absorption region), **b,** Corresponding $|FFT|^2$ spectra

Upon closer examination, as illustrated in Supplementary Figure 31, a noticeable and consistent disparity becomes clear across all sets of dyes and semiconducting molecules, encompassing Squaranines, PBI, BODIPY, Cyanine, PPCy dyes, and NFAs. Even when comparing data points within the same class, multiple instances exist where a lower-bandgap system exhibits nearly identical or even higher PLQEs when compared to its bluer counterpart. Similar observations can be made for the NFAs that were investigated in our work.

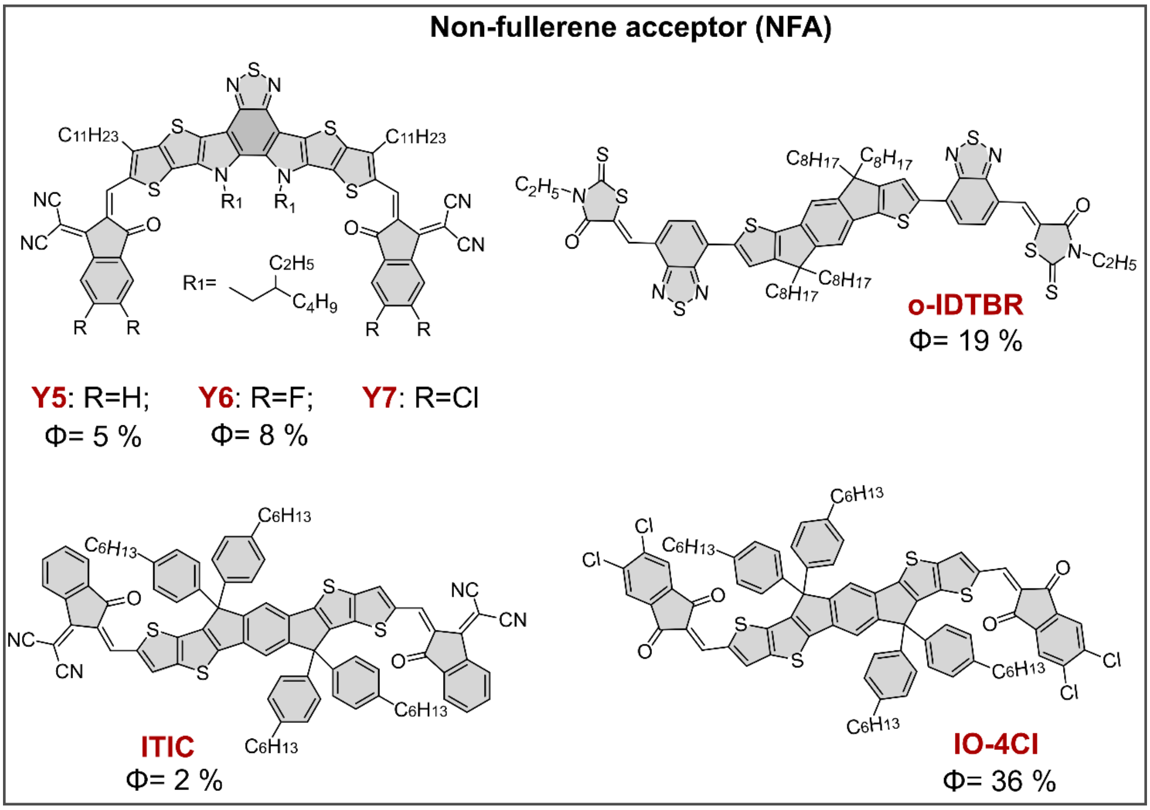

Supplementary Fig. 34: Chemical structures of the Non-fullerene acceptors studied

One key finding is the absence of a significant increase in non-radiative rates when transitioning from IO-4Cl to o-IDTBR and Y6, as depicted in Supplementary Figure 32. This phenomenon can be elucidated by variations in the chemical structures of these molecules, resulting in alterations in intrinsic vibrational frequencies and effective exciton-vibrational coupling, as illustrated in Supplementary Figure 33. Notably, the strongly coupled high-frequency modes in Y5 and Y6 have lower vibrational frequencies compared to IO-4Cl and o-IDTBR, which is the main reason for the similar non-radiative rates observed between IO-4Cl to o-IDTBR and Y6.

References:

1. Gamero, V. *et al.* [4-(N-Carbazolyl)-2,6-dichlorophenyl]bis(2,4,6-trichlorophenyl)methyl radical an efficient red light-emitting paramagnetic molecule. *Tetrahedron Letters* **47**, 2305–2309 (2006).
2. Peng, Q., Blikim Obolda, A., Zhang, M. & Li, F. Organic Light-Emitting Diodes Using a Neutral π Radical as Emitter: The Emission from a Doublet. *Angewandte Chemie* **127**, 7197–7201 (2015).
3. Liebel, M., Schnedermann, C., Wende, T. & Kukura, P. Principles and Applications of Broadband Impulsive Vibrational Spectroscopy. *The Journal of Physical Chemistry A* **119**, 9506–9517 (2015).
4. Schnedermann, C., Liebel, M. & Kukura, P. Mode-specificity of vibrationally coherent internal conversion in rhodopsin during the primary visual event. *Journal of the American Chemical Society* **137**, 2886–2891 (2015).
5. Rafiq, S. & Scholes, G. D. Slow Intramolecular Vibrational Relaxation Leads to Long-Lived Excited-State Wavepackets. *Journal of Physical Chemistry A* **120**, 6792–6799 (2016).
6. Schnedermann, C. *et al.* A molecular movie of ultrafast singlet fission. *Nat Commun* **10**, 4207 (2019).
7. Hosokai, T. *et al.* 58-2: Revealing the Excited-state Dynamics of Thermally Activated Delayed Flourescence Molecules by using Transient Absorption Spectrospy. *SID Symposium Digest of Technical Papers* **47**, 786–789 (2016).
8. Guo, H. *et al.* High stability and luminescence efficiency in donor–acceptor neutral radicals not following the Aufbau principle. *Nat Mater* **18**, 977–984 (2019).
9. Cho, E., Coropceanu, V. & Brédas, J. L. Organic Neutral Radical Emitters: Impact of Chemical Substitution and Electronic-State Hybridization on the Luminescence Properties. *J Am Chem Soc* **142**, 17782–17786 (2020).
10. Ai, X. *et al.* Efficient radical-based light-emitting diodes with doublet emission. *Nature* **563**, 536–540 (2018).
11. Merrick, J. P., Moran, D. & Radom, L. An Evaluation of Harmonic Vibrational Frequency Scale Factors. *The Journal of Physical Chemistry A* **111**, 11683–11700 (2007).

12. Alvertis, A. M. *et al.* Switching between Coherent and Incoherent Singlet Fission via Solvent-Induced Symmetry Breaking. *Journal of the American Chemical Society* **141**, 17558–17570 (2019).

13. Benduhn, J. *et al.* Intrinsic non-radiative voltage losses in fullerene-based organic solar cells. *Nature Energy* **2**, (2017).

14. Zhang, K. *et al.* Theoretical Study of the Mechanism of Aggregation-Caused Quenching in Near-Infrared Thermally Activated Delayed Fluorescence Molecules: Hydrogen-Bond Effect. (2019) doi:10.1021/acs.jpcc.9b06388.

15. Gruhn, N. E. *et al.* The Vibrational Reorganization Energy in Pentacene: Molecular Influences on Charge Transport. *J Am Chem Soc* **124**, 7918–7919 (2002).

16. Regimes of Exciton Transport in Molecular Crystals in the Presence of Dynamic Disorder. https://onlinelibrary.wiley.com/doi/epdf/10.1002/adfm.201503888.

17. Alvertis, A. M. *et al.* Impact of exciton delocalization on exciton-vibration interactions in organic semiconductors. *Physical Review B* **102**, (2020).

18. Yuan, Y. *et al.* Over 10% EQE Near-Infrared Electroluminescence Based on a Thermally Activated Delayed Fluorescence Emitter. *Advanced Functional Materials* **27**, 1700986 (2017).

19. Monserrat, B., Drummond, N. D. & Needs, R. J. Anharmonic vibrational properties in periodic systems: energy, electron-phonon coupling, and stress. *Physical Review B* **87**, 144302 (2013).

20. Abdurahman, A. *et al.* Understanding the luminescent nature of organic radicals for efficient doublet emitters and pure-red light-emitting diodes. *Nat Mater* **19**, 1224–1229 (2020).

21. Hele, T. J. H., Monserrat, B. & Alvertis, A. M. Systematic improvement of molecular excited state calculations by inclusion of nuclear quantum motion: A mode-resolved picture and the effect of molecular size. *Journal of Chemical Physics* **154**, (2021).

22. Yuan, Y. *et al.* Over 10% EQE Near-Infrared Electroluminescence Based on a Thermally Activated Delayed Fluorescence Emitter. *Adv Funct Mater* **27**, 1700986 (2017).

23. Li, C. *et al.* Deep-Red to Near-Infrared Thermally Activated Delayed Fluorescence in Organic Solid Films and Electroluminescent Devices. *Angewandte Chemie International Edition* **56**, 11525–11529 (2017).

24. Lin, Q. *et al.* High-efficiency deep-red quantum-dot light-emitting diodes with type-II CdSe/CdTe core/shell quantum dots as emissive layers. *Journal of Materials Chemistry C* **4**, 7223–7229 (2016).

25. Wang, N. *et al.* Perovskite light-emitting diodes based on solution-processed self-organized multiple quantum wells. *Nature Photonics 2016 10:11* **10**, 699–704 (2016).

26. Tuong Ly, K. *et al.* Near-infrared organic light-emitting diodes with very high external quantum efficiency and radiance. *Nature Photonics 2016 11:1* **11**, 63–68 (2016).

27. Xue, J. *et al.* High-Efficiency Near-Infrared Fluorescent Organic Light-Emitting Diodes with Small Efficiency Roll-Off: A Combined Design from Emitters to Devices. *Advanced Functional Materials* **27**, 1703283 (2017).

28. Gould, I. R., Noukakis, D., Goodman, J. L., Young, R. H. & Farid, S. A quantitative relationship between radiative and nonradiative electron transfer in radical-ion pairs. *J Am Chem Soc* **115**, 3830–3831 (1993).

29. Barbara, P. F., Meyer, T. J. & Ratner, M. A. Contemporary Issues in Electron Transfer Research. *J Phys Chem* **100**, 13148–13168 (1996).

30. Liu, Q. & Vandewal, K. Understanding and Suppressing Non-Radiative Recombination Losses in Non-Fullerene Organic Solar Cells. *Advanced Materials* vol. 35 Preprint at https://doi.org/10.1002/adma.202302452 (2023).

31. Benduhn, J. *et al.* Intrinsic non-radiative voltage losses in fullerene-based organic solar cells. *Nat Energy* **2**, (2017).

32. Azzouzi, M. *et al.* Nonradiative Energy Losses in Bulk-Heterojunction Organic Photovoltaics. *Phys Rev X* **8**, (2018).

33. Jortner, J. Temperature dependent activation energy for electron transfer between biological molecules. *J Chem Phys* **64**, 4860–4867 (1976).

34. Wang, S. F. *et al.* Polyatomic molecules with emission quantum yields >20% enable efficient organic light-emitting diodes in the NIR(II) window. *Nat Photonics* **16**, 843–850 (2022).

35. Wei, Y. C. *et al.* Overcoming the energy gap law in near-infrared OLEDs by exciton–vibration decoupling. *Nat Photonics* **14**, 570–577 (2020).

36. Zewail, A. H. Optical molecular dephasing: principles of and probings by coherent laser spectroscopy. *Acc Chem Res* **13**, 360–368 (1980).

37. Nelson, K. A. & Williams, L. R. Femtosecond time-resolved observation of coherent molecular vibrational motion. *Phys Rev Lett* **58**, 745–745 (1987).

38. Ha, J. M. Y. *et al.* Observation of molecular vibrations in real time. *Phys Rev Lett* **57**, 3302–3302 (1986).

39. Ruhman, S., Joly, A. G. & Nelson, K. A. *Coherent Molecular Vibrational Motion Observed in the Time Domain Through Impulsive Stimulated Raman Scattering*. *IEEE JOURNAL OF QUANTUM ELECTRONICS* vol. 24 (1988).

40. Ghosh, T., Aharon, S., Etgar, L. & Ruhman, S. Free Carrier Emergence and Onset of Electron-Phonon Coupling in Methylammonium Lead Halide Perovskite Films. *J Am Chem Soc* **139**, 18262–18270 (2017).

41. Fu, J. *et al.* Electronic States Modulation by Coherent Optical Phonons in 2D Halide Perovskites. *Advanced Materials* **33**, (2021).

42. Abdurahman, A. *et al.* Understanding the luminescent nature of organic radicals for efficient doublet emitters and pure-red light-emitting diodes. *Nat Mater* **19**, 1224–1229 (2020).

43. Cudazzo, P., Gatti, M. & Rubio, A. Excitons in molecular crystals from first-principles many-body perturbation theory: Picene versus pentacene. *Phys Rev B Condens Matter Mater Phys* **86**, (2012).

44. Alvertis, A. M., Haber, J. B., Engel, E. A., Sharifzadeh, S. & Neaton, J. B. Phonon-Induced Localization of Excitons in Molecular Crystals from First Principles. *Phys Rev Lett* **130**, (2023).

45. Alvertis, A. M. *et al.* Impact of exciton delocalization on exciton-vibration interactions in organic semiconductors. *Phys Rev B* **102**, (2020).

46. Vasilopoulou, M. *et al.* Advances in solution-processed near-infrared light-emitting diodes. *Nature Photonics* vol. 15 656–669 Preprint at https://doi.org/10.1038/s41566-021-00855-2 (2021).

47. Mayerhöffer, U., Gsänger, M., Stolte, M., Fimmel, B. & Würthner, F. Synthesis and molecular properties of acceptor-substituted squaraine dyes. *Chemistry - A European Journal* **19**, 218–232 (2013).

48. Liu, W. *et al.* Low-Bandgap Non-fullerene Acceptors Enabling High-Performance Organic Solar Cells. *ACS Energy Lett* **6**, 598–608 (2021).
